# Bladder and bowel responses to lumbosacral epidural stimulation in uninjured and transected anesthetized rats

**DOI:** 10.1038/s41598-021-81822-3

**Published:** 2021-02-08

**Authors:** Robert F. Hoey, Daniel Medina-Aguiñaga, Fahmi Khalifa, Beatrice Ugiliweneza, Sharon Zdunowski, Jason Fell, Ahmed Naglah, Ayman S. El-Baz, April N. Herrity, Susan J. Harkema, Charles H. Hubscher

**Affiliations:** 1 Department of Anatomical Sciences and Neurobiology, University of Louisville School of Medicine, MDR, 511 S. Floyd St., Room 111, Louisville, KY 40202 USA; 2 Bioengineering Department, University of Louisville J. B. Speed School of Engineering, Louisville, KY USA; 3 Department of Neurological Surgery, University of Louisville School of Medicine, Louisville, KY USA; 4 Kentucky Spinal Cord Injury Research Center, University of Louisville, Louisville, KY USA

**Keywords:** Neuroscience, Gastroenterology, Urology

## Abstract

Spinal cord epidural stimulation (scES) mapping at L5-S1 was performed to identify parameters for bladder and bowel inhibition and/or contraction. Using spinally intact and chronic transected rats of both sexes in acute urethane-anesthetized terminal preparations, scES was systematically applied using a modified Specify 5–6–5 (Medtronic) electrode during bladder filling/emptying cycles while recording bladder and colorectal pressures and external urethral and anal sphincter electromyography activity. The results indicate frequency-dependent effects on void volume, micturition, bowel peristalsis, and sphincter activity just above visualized movement threshold intensities that differed depending upon neurological intactness, with some sex-dependent differences. Thereafter, a custom-designed miniature 15-electrode array designed for greater selectivity was tested and exhibited the same frequency-dependent urinary effects over a much smaller surface area without any concurrent movements. Thus, select activation of autonomic nervous system circuitries with scES is a promising neuromodulation approach for expedient translation to individuals with SCI and potentially other neurologic disorders.

## Introduction

Bladder and bowel dysfunctions, consistently ranked as a top priority issues impacting overall health and quality of life by the spinal cord injury (SCI) population^1,2^, require daily management and toileting programs that most frequently include pharmacological approaches for urinary continence and physical interventions for evacuation (e.g. catheterization for bladder and digital stimulation for bowel). Traditional methods that include pharmacotherapy, non-electrical devices such as catheters, suppositories and irrigation techniques, and surgical procedures when deemed necessary, target management and improve symptomology, but do not replace or restore urinary or bowel control^3^. Techniques targeting function, such as electrical stimulation of spinal nerve roots, peripheral nerves or the peripheral organ itself^4^, have been applied over the years but have not led to widespread clinical use due to limited effectiveness or the irreversible invasive nature of the approach.

Epidural stimulation, which creates an electrical field over the spinal cord, has shown clinical effectiveness for restoration of voluntary and reflex control of the lower urinary tract and bowel^5–10^. For example, early studies investigating the use of percutaneous epidural stimulation in humans found measured improvements in bladder function outlasted the stimulation time^6^ and occurred regardless of spinal cord disease etiology^6^. Furthermore, application of spinal cord epidural stimulation (scES) is effective in a large proportion of recipients (77.5% of 40 multiple sclerosis patients^5^, for example). Our initial scES study in five male research participants targeting the bladder revealed significantly greater emptying with scES than without, as well as significantly greater efficiencies of voiding with higher (30, 45 and 60 Hz) than with lower (5 and 10 Hz) frequencies at intensities just below motor threshold^8^.

For the current study, systematic mapping experiments of L5-S1 spinal cord were conducted in rats (intact and chronic transected—T9 spinal cord level) as an important initial step toward identifying optimal scES stimulation parameters and key elements to inform potential underlying mechanisms. L5-S1 was targeted with scES first as this spinal level has inputs/outputs of pelvic (parasympathetic) and pudendal (somatic) nerves supplying the pelvic visceral organs (equivalent to S2-4 in humans). Several pre-clinical studies using the rodent model have shown effects of scES on external urethral sphincter (EUS) activity^11–13^. Numerous variables examined in the current study allowed for multiple novel key comparisons, including scES effects upon intact versus chronic spinal transection conditions, male versus female responsiveness, holding versus emptying outcomes, influence of stimulus intensity and frequency combinations on pelvic organ function and visualized movements (VisMvt), and lower urinary tract versus lower gastrointestinal tract physiological processes. The scES mapping results were then used to guide a pilot study regarding the effectiveness and selectivity of a custom-designed multi-electrode array for use in rodents.

## Results

**Figure 1:**
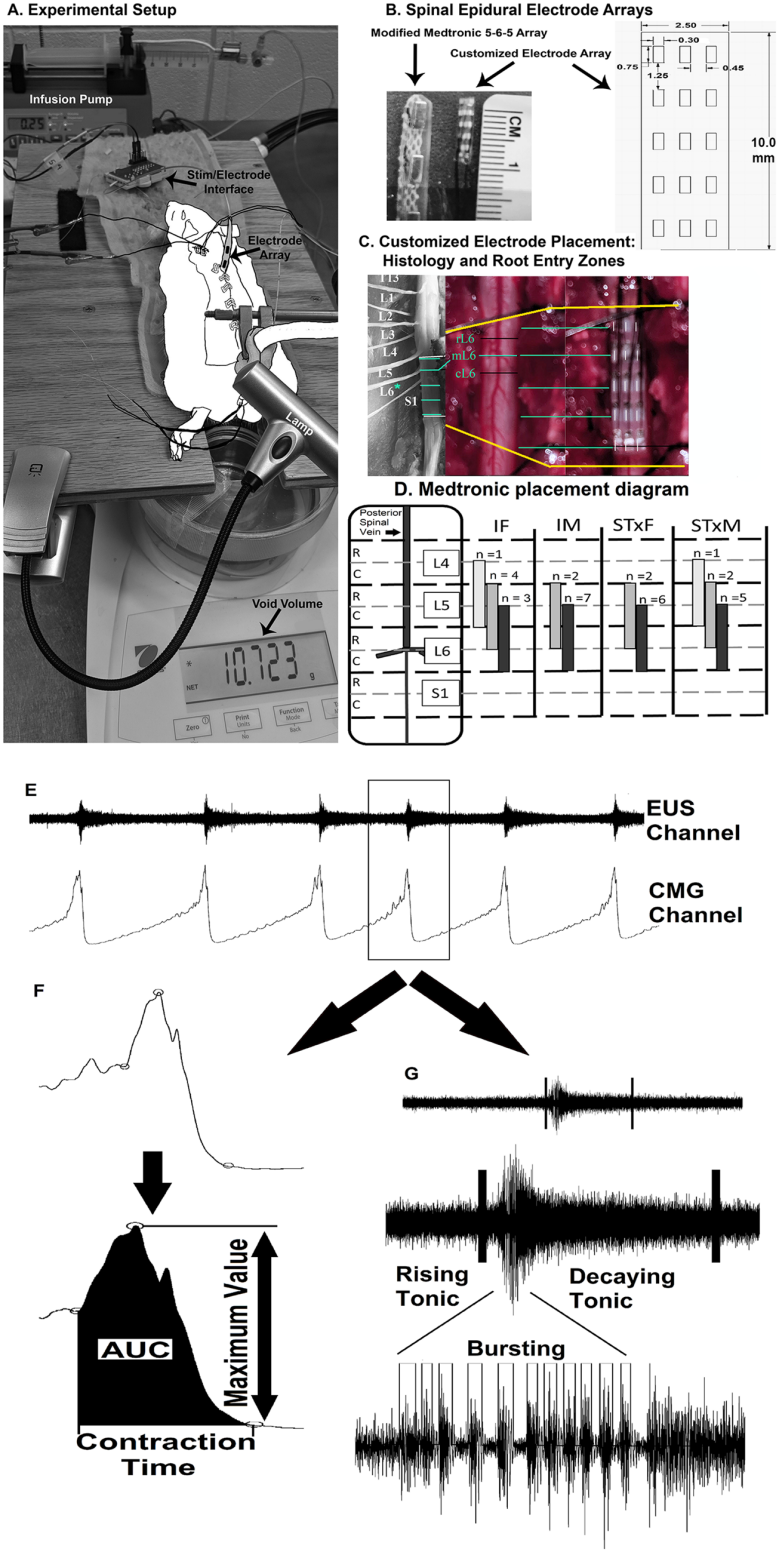
Experimental setup, electrode design/placement, and data analysis. (**A**) Experimental setup showing the rat on a raised platform to accommodate the balance for measuring void volume. The infusion pump (set at 0.25 ml/min) infuses saline into the bladder via a catheter in the bladder dome. (**B**) A modified Medtronic 5–6–5 array is shown alongside a customized Micro-Leads array. The diagram (right side) depicts the custom array (scale is mm). (**C**) Spinal level of electrode placement is determined after perfusion by markings (suture knots in overlaying muscle) and root level (shown and labeled in left image). *Note*: spinal levels are operationally defined as rostral (portion about the dorsal root entry zone) and caudal (below dorsal root entry zone). (**D**) Schematic representation of Medtronic electrode placement in all animals. *R* rostral, *C* caudal. Quantification of data was accomplished with a customized program. This program takes the cystometrogram (CMG) bladder pressure traces (bottom trace, **E**, labelled CMG channel) and places start and end markers for each void contraction (**F**, indicated by circles). Maximum contractile pressure, area under the curve, contractile time, and inter-contractile interval can then be recorded (**F**). The electromyography (EMG) channel from the external urethral sphincter (EUS; top trace, **E**) is analyzed by first comparing the amplitude to an initial baseline period. When the amplitude is twice the baseline amplitude it is considered the beginning of activity, whereas when activity returns back below this threshold it is the end of activity. During this activity period there are three divisions: rising tonic **(**from onset to bursting period), bursting time (**G**, bursts of activity with quiescent periods in between), and decaying tonic (from end of bursting to end of activity). Once characterized, the amount of time in each phase, amplitude, bursting frequency, pulse width, and inter-burst interval can be recorded.

The L5-S1 Medtronic array placement for each group of animals tested is provided with the experimental setup diagram (Fig. 1D). All reported results come from use of the Medtronic array only, unless otherwise stated. Individual data for 33 animals, those that completed the entire testing array (intact female: n = 8; intact male: n = 9; transected female: n = 8; transected male: n = 8), were averaged together by group for pre-mapping baseline outcomes, for each of 30 parameter combinations tested, and for post-mapping baseline outcomes. Note that due to individual differences in sensitivity, not all animals received the highest intensity (500 µA) of scES as a widespread VisMvt response often occurred with scES at 300 µA. Heat maps were generated from mean group data for each of 15 quantified cystometrograms (CMG), sphincter electromyography (EMG), and anorectal manometry (ARM) outcome measures (see Methods and Fig. 1E,F,G). For a translational perspective, data was reduced to functionally appropriate components encompassing lower/higher frequencies of stimulations^14,15^ and below/above VisMvt stimulation intensities (see data below) for the parameters tested in order to provide a functional comparison with scES parameters used in our current, parallel human study. Four data quadrants for analysis (divisions shown by black dividing lines on heatmaps) included: below VisMvt intensity at low frequencies (Quadrant 1; 50–150 µA, 5–10 Hz), below VisMvt at high frequencies (Quadrant 2; 50–150 µA, 30–60 Hz), above VisMvt at high frequencies (Quadrant 3; 300–500 µA, 30–60 Hz), and above VisMvt intensity at low frequencies (Quadrant 4; 5–10 Hz, 300–500 µA). Mapping results are summarized below with illustrations showing typical examples. Statistical data for all quadrant group comparisons for each outcome measure is provided in Tables 1 (urinary, rats having bladder contractions), 2 (urinary, acontractile rats—absence of contractions) and 3 (bowel). Although scES effect patterns (i.e. bladder changes) observed were generally similar between female and male rats, distinct sex differences were evident (discussed in outcome measure categories below).

**Table 1:** Bladder outcome measures. Significant differences for outcome measures reflecting bladder (cystometry-CMG) and external urethral sphincter (electromyography-EMG) activity with (scES ON) and without (scES OFF) epidural stimulation. The EMG section contains blank cells as those comparisons could not be assessed due to lack of response.

	Intact male vs female		Intact vs transected female	
**Bladder dynamics outcomes**	**scES ON**			
Void volume (cc)	OA, Q2	IF > IM	OA, Q1, Q2; Q3	IF > STxF; STxF > IF
Intercontraction interval (sec)	N.S	–	Q3	IF > STxF
Max pressure (mmHg)	OA, Q4	IM > IF	Q3	IF > StxF
CMG AUC (mmHg s)	N.S	–	Q3	IF > STxF
	**scES OFF**			
Void volume (cc)	N.S	–	OA, Q1-4	IF > STxF
Intercontraction interval (s)	N.S	–	N.S	–
Max pressure (mmHg)	N.S	–	N.S	–
CMG AUC (mmHg s)	N.S	–	N.S	–
**Electromyography outcomes**	**scES ON**			
EMG total time (s)	OA, Q1-4	IF > IM	OA, Q1-2	STxF > IF
Mean burst time (s)	OA, Q1-3	IM > IF		
Burst freq	OA, Q1-2	IF > IM		
Burst ratio	Q2	IF > IM		
Burst time (s)	OA, Q1-4	IM > IF		
	**scES OFF**			
EMG total time (s)	OA, Q1-4	IF > IM	OA, Q1-2	STxF > IF
Mean burst time (s)	OA, Q1-3	IM > IF		
Burst freq	OA, Q1-4	IF > IM		
Burst ratio	OA, Q1,3,4	IF > IM		
Burst time (s)	OA, Q1-4	IM > IF		

*OA* overall significant difference (regardless of quadrant), *N.S.* no significant differences, *Q* quadrant number from heatmap analysis (divisions as illustrated in Fig. 4 for volume map), *IM* intact male, *IF* intact female, *STxF* transected female, *CMG AUC* cystometrogram area under the curve. > reflects direction of significant difference.

**Table 2:** Bladder outcome measures for non-voiding phenotype. Separate quantification for transected rats with a lack of bladder contractions during cystometrogram recordings.

	Within STxF	Within STxM	Trasected male versus female	
**Non-voiding phenotype**	**scES ON**			
AUC (mmHg s)	N.S	N.S	OA, Q3	STxM > STxF
Mean pressure (mmHg)	Q1,2 > Q3,4; Q4 > Q3	N.S	OA, Q1-4	STxM > STxF
Max pressure (mmHg)	Q1,2 > Q4; Q3 > Q1,2,4	Q3 > Q1,2; Q4 > Q1,2	OA, Q1-4	STxM > STxF
Min pressure (mmHg)	Q1 > Q3,4; Q2 > Q3; Q4 > Q3	Q2 > Q3	OA, Q1-4	STxM > STxF
	**scES OFF**			
AUC (mmHg s)	N.S	Q4 > Q1-3	OA, Q2,4	STxM > STxF
Mean pressure (mmHg)	Q1,2 > Q3,4	N.S	OA, Q1-4	STxM > STxF
Max pressure (mmHg)	Q1 > Q4	Q4 > Q1,2,3	OA, Q1-4	STxM > STxF
Min pressure (mmHg)	Q1,2 > Q3,4 Q4 > Q3	Q2 > Q3,4	OA, Q1-4	STxM > STxF
**Comparison of scES ON versus OFF**	**STxF**	**STxM**		
AUC (mmHg s)	N.S	OA, Q3,4 OFF > ON		
Mean pressure (mmHg)	Q3 OFF > ON	N.S		
Max pressure (mmHg)	OA ON > OFF; Q3 ON > OFF	OA ON > OFF; Q3 ON > OFF		
Min pressure (mmHg)	Q3 OFF > ON	Q4 ON > OFF		

*AUC* area under the curve, *OA* overall significant difference (regardless of quadrant), *N.S.* no significant differences, *Q* quadrant number from heatmap analysis (divisions as illustrated in Fig. 4 for volume map), *STxF* transected female, *STxM* transected male. > reflects direction of significant difference.

### Bladder function results

Baseline uninjured, pre-mapping bladder fill-empty cycle data demonstrated consistent intra-animal void volumes (thus 5 cycles were averaged together); however, there was high inter-animal variability. Intact female and male rats had similar baseline micturition volume data (Intact female- mean = 0.596 cc, SEM = 0.05; Intact male—mean = 0.587 cc, SEM = 0.08), whereas transected rats showed slightly lower volumes between males and females (Transected female—mean = 0.460 cc, SEM = 0.04; Transected male—mean = 0.395 cc, SEM = 0.03). When pre-test and post-test baseline values were averaged, intact females (0.643 cc, SEM = 0.05) had a significantly greater mean baseline volume than transected males (0.375 cc, SEM = 0.06).

**Figure 2:**
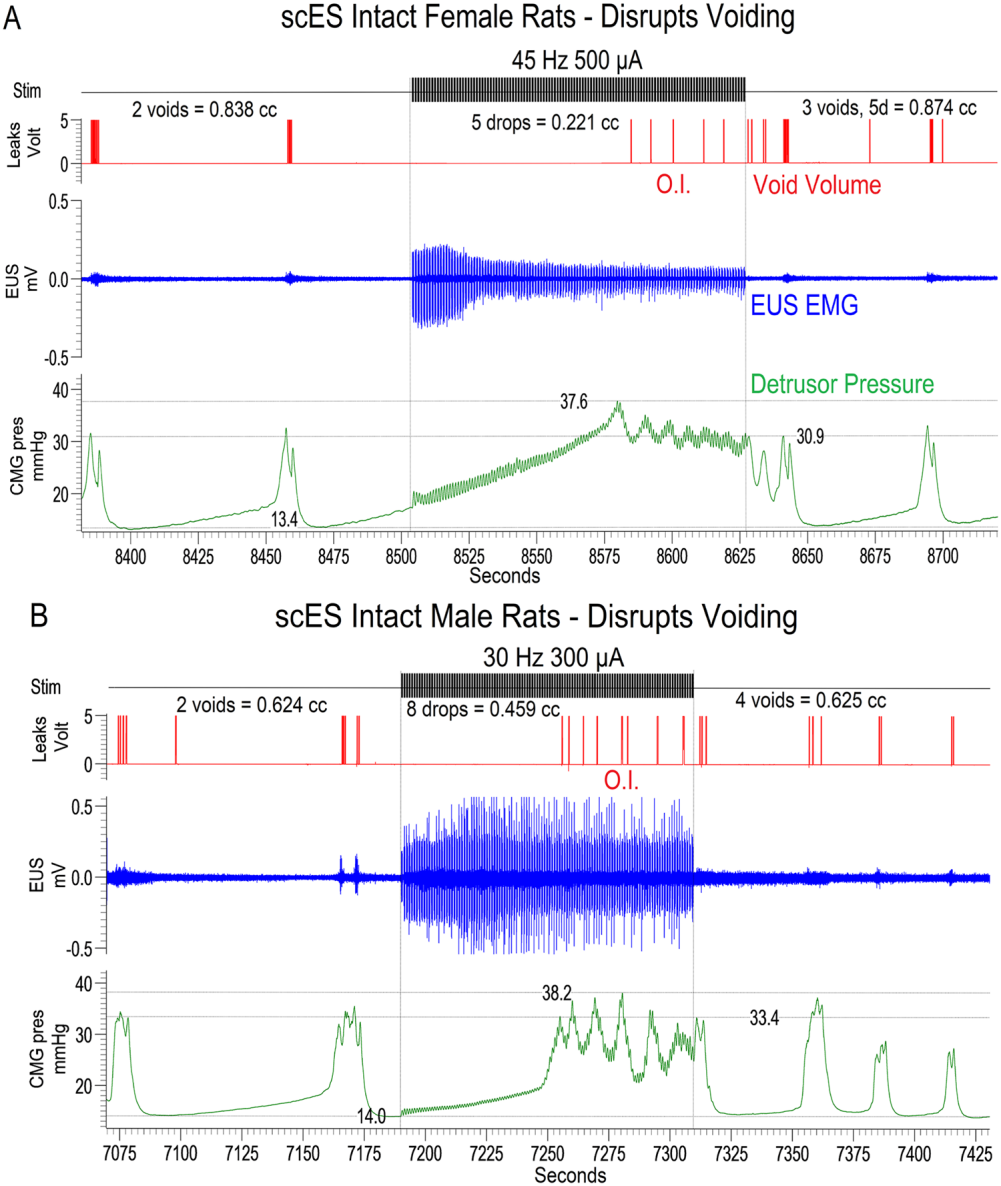
Typical examples of inhibition of voiding with scES at L5-S1 in intact males and females. Intact animals, both female **(A**) and male **(B**), have a steep rise in pressure until overflow incontinence (O.I.) occurs, but no void contraction of the bladder during spinal cord epidural stimulation (scES). A void occurs upon offset of scES (end of vertical marker). Thus, a *storage-like *effect **(A**,**B**) is generated by scES at L5-S1 in spinally *intact* male and female rats, as evidenced by suppression of voiding during stimulation with enlarged area under the curve, contraction time and higher peak pressure, followed by bladder contraction accompanied by EUS bursting and a void at stimulus offset. These effects on bladder function in intact rats are both frequency and intensity dependent and reflect known circuitries^23^.

**Figure 3:**
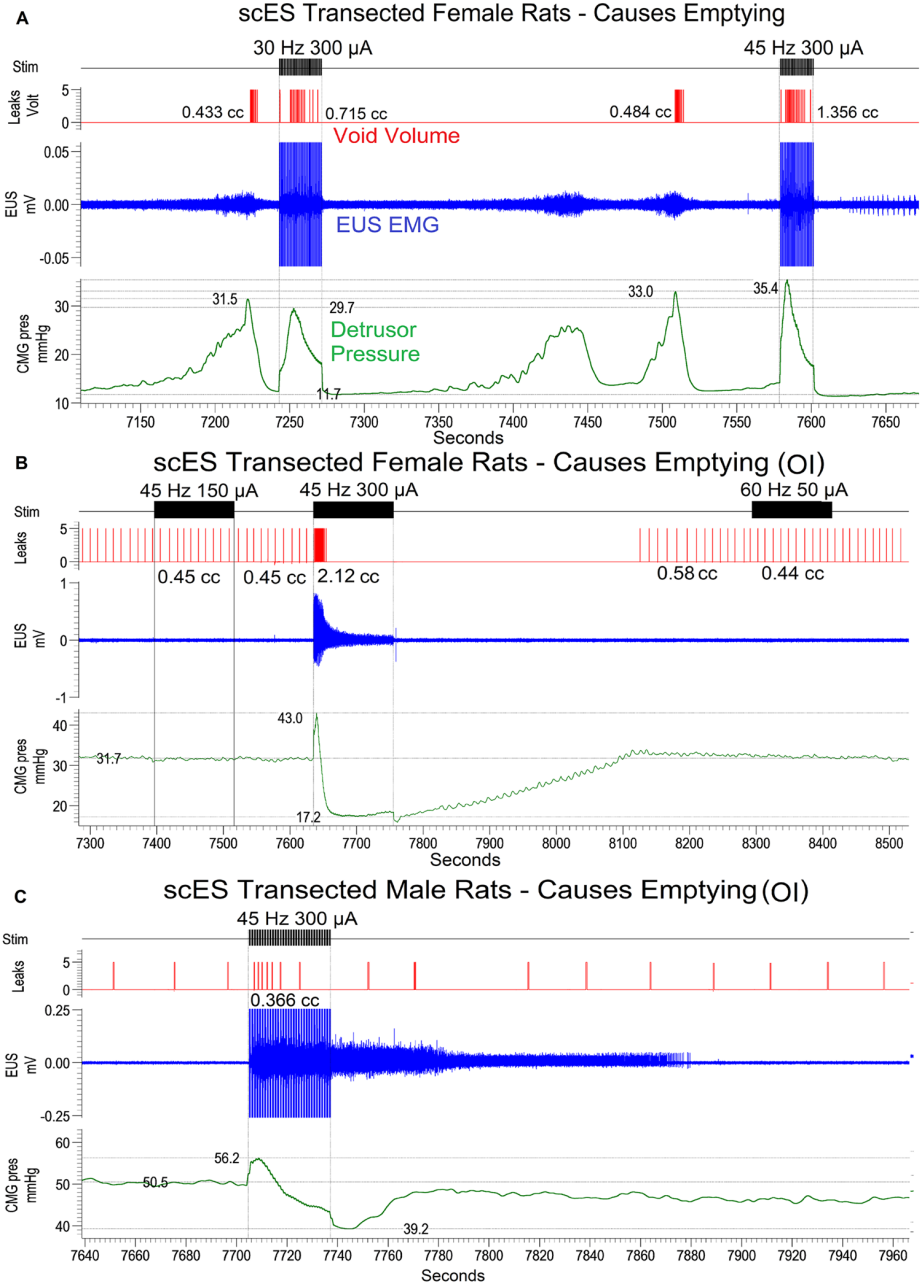
Typical voiding responses induced with scES in T9 transected males and females. After chronic transection, two patterns of fluid expulsion were found: voiding with EUS bursting (females only, **A**) and overflow incontinence (females, **B**; males, **C**). Regardless of phenotype, all transected animals empty immediately at the onset of scES. A contraction is triggered shortly after onset of scES using above-MT intensity at L5-S1, as evidenced by reduced area under the curves, maximum pressures and shorter more efficient contraction times with larger volumes voided (see heat maps in Fig. 4).

In addition, several CMG patterns were observed prior to mapping with scES. Most intact female and male rats had low filling pressures and steep rises in detrusor pressure upon reaching capacity and exhibited typical micturition pressure curves with concomitant EUS bursting during the emptying phase followed by a post-void decrease in detrusor pressure to baseline (see Fig. 2A,B). A subset of three intact males (33%; 0% of intact females) went into overflow incontinence upon reaching capacity during the first fill cycle and remained that way throughout the experimental testing session (continuous fluid expulsion at an elevated detrusor pressure, Fig. 2). In contrast, among the chronic transection groups (n = 16), 40% of females and 100% of males went into overflow incontinence (OI) at the start of cystometry (Fig. 3B,C). Although these occurrences could transpire if the plane of urethane anesthesia is too deep, the sex and intact/transection group differences suggest other factors were likely involved (see “Discussion”).

**Figure 4:**
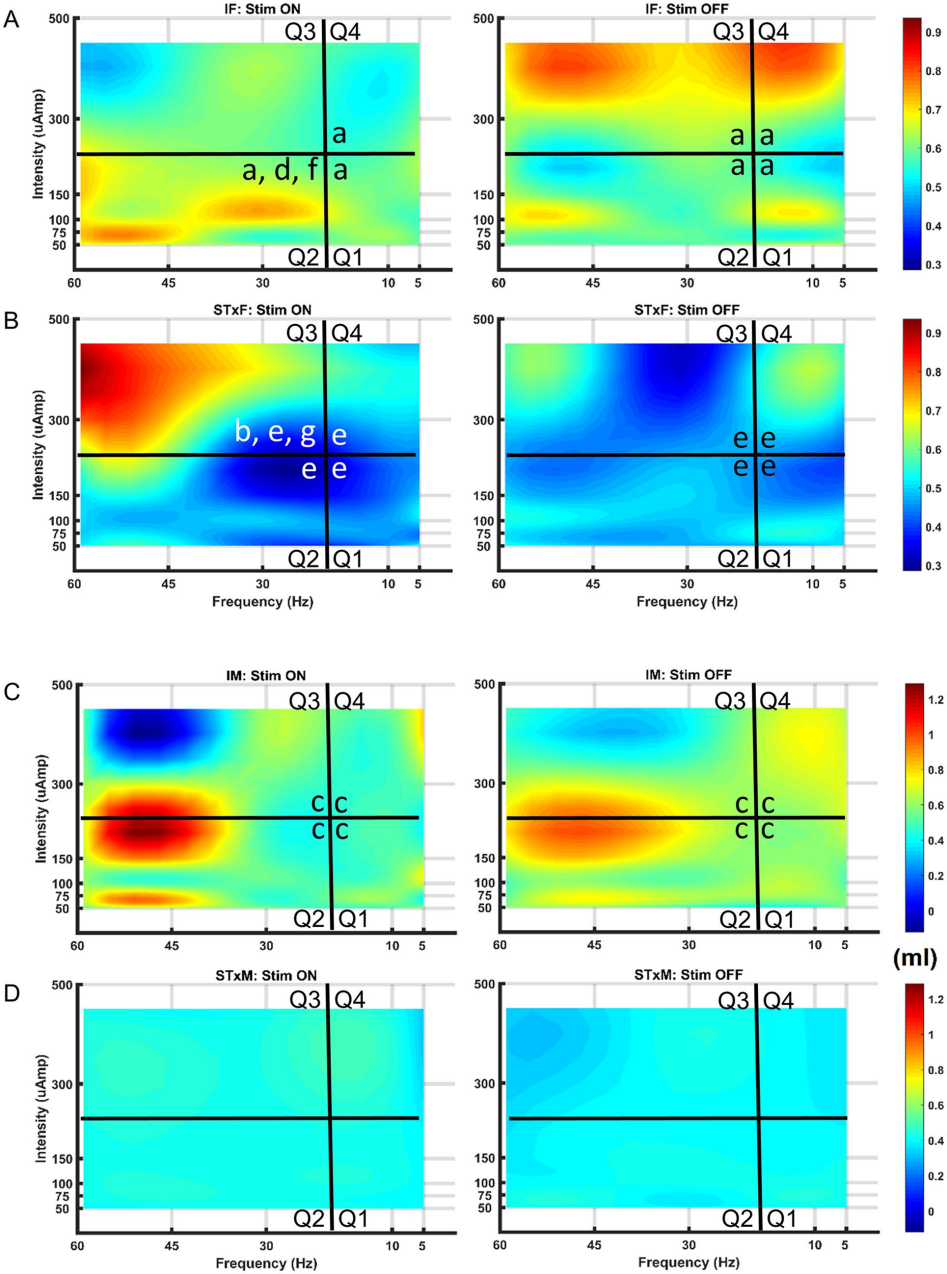
Summary heat maps illustrating micturition changes due to spinal cord epidural stimulation (scES). Heat maps showing void volumes (ml) from all intact female (IF, **A**), T9 transected female (STxF, **B**), intact male (IM, **C**), and T9 transected male (STxM, **D**) groups of Wistar rats. Void volume data with scES (Stim ON, left column) and without scES (Stim OFF, right column) at L5-S1 are provided. Each map is subdivided into low/high frequency and intensity quadrants (Q1-4) with micturition volume ranges indicated to the far right (dark blue—low; dark red- high). Intact female rats (**A**) have a reduced volume due to suppression of the bladder to contract with Stim ON (left Q3; higher intensities and frequencies), although some volume is still generated due to overflow incontinence. A bladder contraction and void occur upon offset of scES (right side of **A**, Q3), reflected by the larger volumes. At lower intensities but higher frequencies (Q2), significantly higher volumes are generated, relative to Stim OFF, other Stim ON parameters (Q1,3,4), and the transected female group (Q2, **B**). Unlike the IF group, STxF animals void immediately at the onset of higher intensity and frequency scES (Q3 in **B**). Note that the higher micturition volumes post-transection (**B**, Q3) reflect over-distended bladders. The same general patterns were seen for males and the low micturition volumes in male rat’s post-transection reflects the acontractile condition (presence of overflow incontinence; see “Discussion”). Overall group void volume differences, regardless of frequency/intensity, were IF > STxF (for both Stim ON and OFF), IM > STxM (for both Stim ON and OFF), IF > IM (Stim ON only) and STxF > STxM (Stim ON and OFF). These differences reflect larger volumes for intact relative to injured animals regardless of sex, and greater void volumes in females. Between quadrant group statistical differences demarcated by placement of a black or white lowercase letter in the greater area are (a) IF > STxF; (b) STxF > IF; (c) IM > STxM; (d) IF > IM; and (e) STxF > STxM. Within group differences are (f) Q2 > Q1,3 and Q2 On > Off; (g) Q3 > Q1,2,4 and Q3 On > Off.

During mapping with the Medtronic electrode (regardless of CMG pattern per above), micturition was modulated with scES in both intact female and male rats. In females, scES induced a storage-like effect (a hold when the bladder was full), as illustrated in Fig. 2A typical example showing overflow incontinence and a lack of contraction upon reaching capacity and as summarized in the excreted volume heat maps (Fig. 4A in Quadrant 3 by blue shadings (low volume) with scES ON at VisMvt intensities with high frequency stimulation versus OFF (red color in right heat map—high volume)). In addition, an increase in void volume was found when below VisMvt intensities were paired with high frequency (i.e. 50–150 µA, 30–60 Hz) scES (Quadrant 2; yellow-orange ON versus blue OFF; Fig. 4A). Note that the significant elevation of void volume in Quadrant 3 of the heat map (Fig. 4A) during the OFF period reflects a post-hold rebound (short latency rebound contraction; see example in Fig. 2A). In intact males (Fig. 4C heat map), although the same patterns were observed in Quadrants 2 and 3 (see typical example in Fig. 2B), the ON/OFF comparison was not statistically significant, which could reflect a post-scES carry-over effect into the OFF period (less of a post-hold rebound effect). An overall sex difference for all quadrant volume data combined with scES ON (void volumes higher in females during stimulation) suggests fundamental differences in male and female micturition ability. One plausible explanation, apparent throughout testing in males, is that scES generated seminal plug material which likely resulted in partial urethral obstruction, thus contributing to lower micturition volumes (see further in “Discussion”).

When stimulation was ON, intact males had greater maximum pressure than intact females even though less volume was produced (Supplemental Table 1 and 2). With scES OFF, there was no longer a sex difference in the measured outcomes. The difference may be due to how voiding is accomplished between male and female rats^16^, and stimulation causing EUS activity that leads to higher vesicle pressure during a void and limiting fluid flow.

For the transected groups of animals, transected females responded to above-VisMvt intensity at high frequency scES (i.e. 300–500 µA, 30–60 Hz) with an immediate void (Quadrant 3, Fig. 4B). A representative trace is provided in Fig. 3A. Similar patterns were observed for transected males (Fig. 4D), although as with intact groups, statistical significance was not reached. Importantly, these effects occurred regardless of presence/absence of bladder contractions (see Fig. 3B,C), suggesting the release of urine was not due to ancillary gross motor movements that occurred with the higher intensity levels of stimulation (above VisMvt; see last section of Results). Rather, scES initiated a detrusor muscle contraction after chronic transection. These same stimulation parameters suppressed bladder contractions in the absence of transection, reflecting injury-induced plasticity of the circuitries (see “Discussion”).

Voided volumes were always greater in transected female than male rats, whether stimulation was ON or OFF (see Fig. 4, B versus D), reflecting fundamental differences in the voiding capability between sexes even after the same injury. Note that animals that did not have bladder contractions (transected male—100%; transected female—33%) were analyzed separately for several CMG parameters (Table 2). For example, transected males had greater overall AUC, mean pressure, peak pressure, and baseline pressure than transected female animals. These findings likely reflect the greater volume expelled by transected females in both the ON and OFF conditions. Transected male animals had the lowest expelled volume of all groups and therefore represents the group with the most inefficient, and most compromised, bladder function. Typical void amounts, reflected by the light blue color (Fig. 4D), are roughly 0.5 cc’s, the infused volume expected during 2 min of filling at a rate of 0.25 cc per minute (continuously filling while at capacity; thus, the amount that was infused was excreted out).

### EUS EMG results

Both transected groups had impaired EUS EMG responses, therefore EMG traces could not be fully quantified. Sixty-six percent of transected female animals had voiding activity with increased EMG activation during voids, but this activity did not contain any bursting activity. Therefore, only total EMG activity time was measured, as changes in intensity across time at different frequencies could not be discriminated. Furthermore, the non-voiding phenotypes in transected male rats was coupled with an impaired EMG signal.

For intact groups, females had significantly longer total EMG activity time in all quadrants (both scES ON and OFF). However, transected female rats had longer EMG activation times with below-VisMvt than with above-VisMvt intensity stimulation (Q1, 2 > Q3, 4), suggesting an intensity but not frequency dependent effect on EUS circuitry after chronic transection. A comparison of intact female, intact male, and transected female rats shows that total EMG activity time was greatest in transected females, intermediate in intact female, and shortest in intact male rats (Table 1). Analysis of the bursting components shows that intact male rats had the greatest average bursting time (sec) and bursting time (sec) but no difference in the bursting frequency (Hz) or bursting:tonic ratio. This finding suggests that the reduced total activity time in intact male rats is due to the longer bursting activity facilitating fluid expulsion.

### Bowel function results

**Figure 5:**
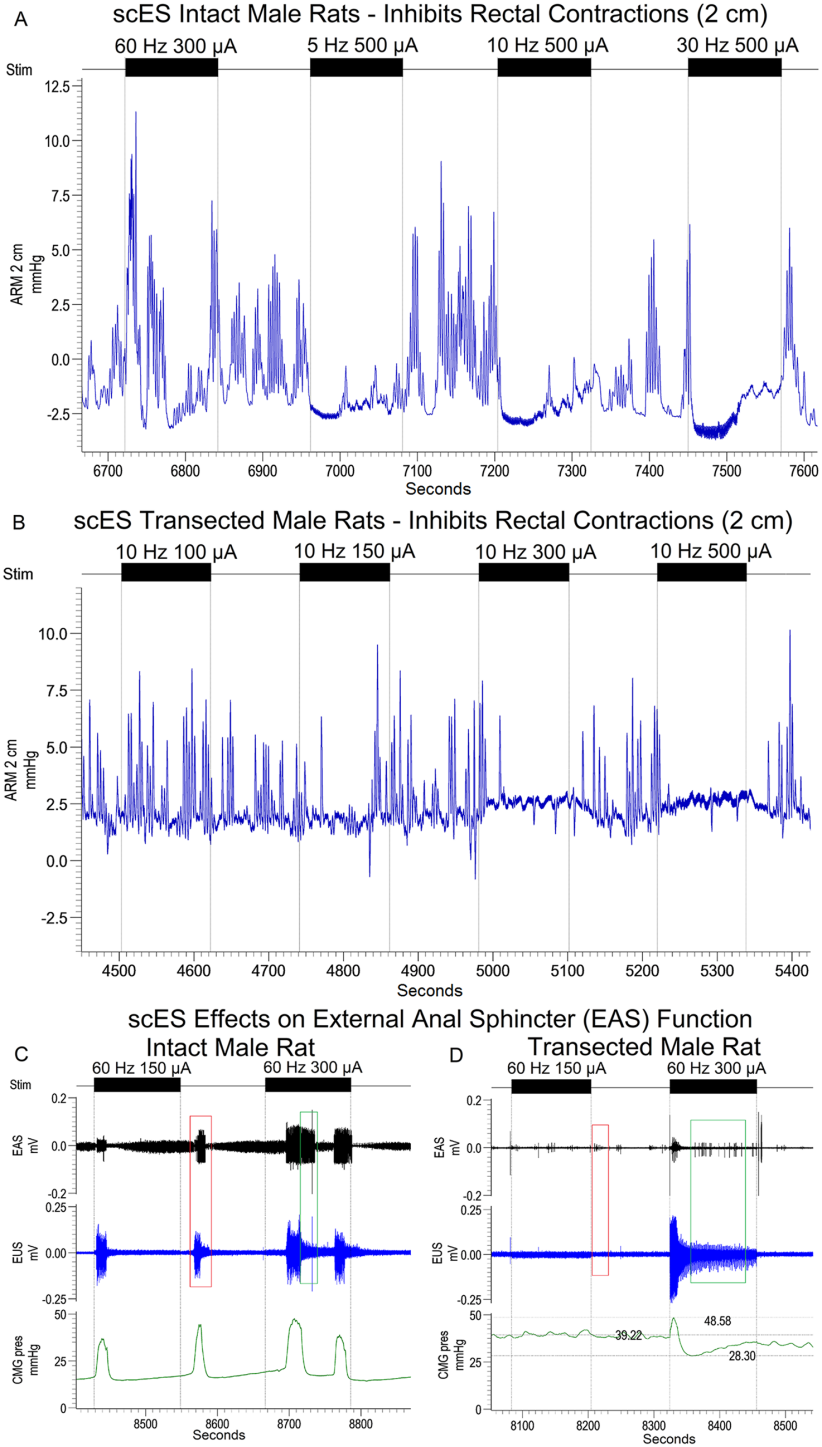
Inhibition of rectal contractions by high intensity scES stimulation and external anal sphincter activity. scES caused a similar reduction in contractions in all groups. Shown are the Intact male (**A**) and Transected male (**B**) groups with a range of intensities from sub-optimal (**A**, 60 Hz 300 µA; **B**, 10 Hz 100 µA) which does not fully inhibit ongoing peristalsis to complete suppression of activity. Background tonic activity and irregular bursting comprises the majority of EAS activity (**C**,**D**). Some intact animals (proportions in results) showed coincident EAS and EUS activity (red box, **C**) which was absent in all animals following transection (red box, **D**). Furthermore, high intensity stimulation, similar to that which inhibits peristalsis, was able to promote EAS activity and lengthen the bouts of activity (green box, **C**) which rarely occurred after transection (green box, **D**).

**Figure 6:**
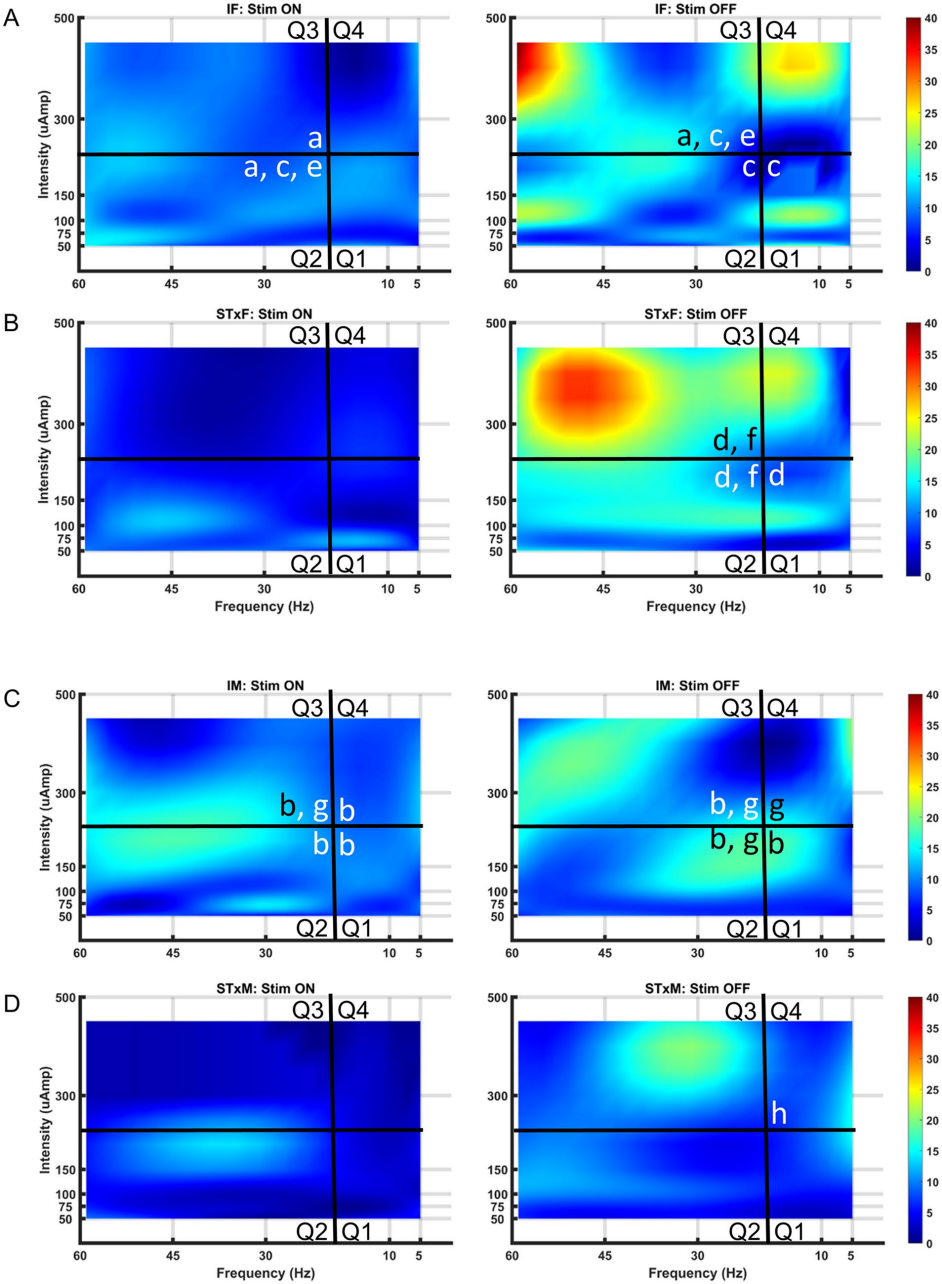
Summary heat maps showing counts of peristaltic waves within a contractile bout at 2 cm depth (rectum) for all groups. Heat map colors reflecting number of contractions are divided into 4 quadrants (Q1-4) and represent data from all intact female (IF, **A**), T9 transected female (STxF, **B**), intact male (IM, **C**), and T9 transected male (STxM, **D**) groups of Wistar rats. The response of the rectum (2 cm from anal verge) was similar across groups with lower activity during scES (left plot of all sections) than when stimulation was off (right plot of all sections). Furthermore, at high scES frequencies and intensities (Q3, upper left quadrants), a rebound effect occurred in the OFF period characterized by a high number of contractions (shown by red color). This finding suggests that the low number of contractions during stimulation is not just an absence of activity but an inhibition that is followed by disinhibition. Overall group differences in counts, regardless of frequency/intensity, were IF > STxF (for both Stim ON and OFF), IM > STxM (for both Stim ON and OFF), IF > IM (Stim OFF only) and STxF > STxM (Stim ON and OFF). These differences reflect greater counts of peristaltic waves for intact relative to injured animals regardless of sex, and greater counts in females. Between quadrant group statistical differences demarcated by placement of a black or white lowercase letter in the greater area are (a) IF > STxF; (b) IM > STxM; (c) IF > IM; and (d) STxF > STxM. Within group differences are (e) STIM ON—Q2 > Q1,4 and STIM OFF—Q3 > Q1,2,4; (f) Off > On Q2,3; (g) STIM ON—Q3 > Q1 and STIM OFF—Q2,3,4 > Q1; and (h) Off > On Q4.

Manometry (ARM) traces were quantified for numerous outcomes including amplitude, AUC, duration, range, contraction count (within bouts), contraction count (non-bout), contraction frequency, and maximal contraction amplitude at both 2 cm (rectum) and 10 cm (distal colon) depths. In most cases, and with most variables, the overall effect of L5-S1 scES was to reduce bowel peristaltic wave activity (see Fig. 5 A,B for trace examples and Fig. 6 for contractile count heat maps). The only exception to this rule were: maximum amplitude (2 cm, Q3, intact male), mean AUC (2, cm, Q1, transected male), and contraction frequency (10 cm, Q2 and Q3, transected male and transected female). This reduction is evidenced by within group statistical differences showing that contractile counts (threshold of twice baseline pressure) with scES OFF was almost always greater than scES ON (full comparisons in Supplemental Tables 3 through 6-AUC, duration, bout count, frequency, maximum amplitude, non-bout count). Importantly, the reduction in contraction counts (within and outside bouts), maximal amplitude, duration, and frequency shows suppression of nearly all contractile dynamics due to scES.

**Table 3:** Bowel outcome measures. Outcome measurements for bowel function data collected from pressure probes inserted to 2 (rectum) and 10 (distal colon) cm from the anal verge depths.

	Intact		Transected		Female		Male	
**Rectum 2 cm**	**scES ON**							
Mean amplitude (mmHg)	OA, Q1-4	IM > IF	OA, Q1,2	STxF > STxM	OA, Q1-4	STxF > IF	OA, Q3	IM > STxM
Max amplitude (mmHg)	OA, Q2,3,4	IM > IF	OA, Q1,2,4	STxF > STxM	OA, Q1,2,4	STxF > IF	OA, Q2,3,4	IM > STxM
Mean AUC (mmHg s)	OA, Q1-2; Q3	IF > M; IM > IF	OA, Q1,2,3	STxM > STxF	Q1	STxF > IF	OA, Q1-4	STxM > IM
Mean duration (sec)	OA, Q1,2,4	IF > M	OA, Q1-4	STxM > STxF	OA, Q1,2,4	IF > STxF	OA, Q1-4	STxM > IM
Mean range	OA, Q2,3,4	IM > IF	OA, Q1,2	STxF > STxM	OA, Q1-4	STxF > IF	Q3	IM > STxM
Count bout	Q2	IF > IM	OA	STxF > STxM	OA, Q2,3	IF > STxF	OA, Q1-4	IM > STxM
Count non-bout	N.S	-	N.S	–	N.S	–	Q2	STxM > IM
Contraction freq	OA, Q2	IF > M	OA, Q1-4	STxF > STxM	OA, Q2	IF > STxF	OA, Q1-4	IM > STxM
**Rectum 2 cm**	**scES OFF**							
Mean amplitude	OA, Q1-4	IM > IF	OA, Q1-3	STxF > STxM	OA, Q1-4	STxF > IF	Q3	IM > STxM
Max amplitude	OA, Q2	IM > IF	OA, Q1-3	STxF > STxM	OA, Q1-3	STxF > IF	OA	IM > STxM
Mean AUC	OA, Q1,2,4	IF > IM	N.S	–	OA, Q1,2,3	STxF > IF	OA, Q1-4	STxM > IM
Mean duration	OA, Q1-4	IF > IM	OA, Q1-4	STxM > STxF	OA, Q1,2	IF > STxF	OA, Q1-4	STxM > IM
Mean range	OA, Q1-4	IM > IF	OA, Q1-3	STxF > STxM	OA, Q1-4	STxF > IF	N.S	–
Count bout	OA, Q1-3	IF > IM	OA, Q1-3	STxF > STxM	OA, Q3	IF > STxF	OA, Q1-3	STxM > IM
Count non-bout	OA, Q3,4	IF > IM	N.S	–	N.S	–	N.S	–
Contraction freq	OA, Q1-3	IF > IM	OA, Q1-3	STxF > STxM	N.S	–	OA, Q1	IM > STxM
**Distal colon 10 cm**	**scES On**							
Mean amplitude	OA, Q1,3,4	IM > IF	N.S	–	OA, Q1	STxF > IF	OA, Q1,2	IM > STxM
Max amplitude	OA, Q1,2,4	IM > IF	N.S	–	Q1	STxF > IF	OA, Q1,2	IM > STxM
Mean AUC	OA, Q1-4	IM > IF	OA, Q1,2	STxM > STxF	OA, Q1,2	STxF > IF	OA, Q3,4	IM > STxM
Mean duration	OA, Q1-4	IM > IF	OA, Q1-4	STxM > STxF	OA, Q1	IF > STxF	Q3,4; Q1	IM > STxM; STxM > IM
Mean range	OA, Q1,2,4	IM > IF	N.S	–	OA, Q1	STxF > IF	N.S	–
Count bout	Q1	IM > IF	OA, Q1	STxM > STxF	N.S	–	N.S	–
Count Non-bout	OA, Q2	IM > IF	OA, Q2,3	STxM > STxF	OA, Q1-4	IF > STxF	OA, Q1,2,4	IM > STxM
Contraction Freq	OA, Q1,2,4	IM > IF	N.S	–	OA, Q1-4	STxF > IF	N.S	–
**Distal colon 10 cm**	**scES OFF**							
Mean amplitude	OA, Q1,2	IM > IF	N.S	–	Q2	STxF > IF	OA, Q1,2	IM > STxM
Max amplitude	OA, Q2	IM > IF	N.S	–	N.S	–	OA, Q1,2	IM > STxM
Mean AUC	OA, Q1-4	IM > IF	OA, Q1,2	STxM > STxF	OA, Q2,3	STxF > IF	OA	IM > STxM
Mean duration	OA, Q1-4	IM > IF	OA, Q1-4	STxM > STxF	OA, Q4	IF > STxF	Q4	IM > STxM
Mean range	OA, Q1,2,4	IM > IF	N.S	–	Q2	STxF > IF	N.S	–
Count bout	Q1; Q3	IM > IF; IF > IM	OA, Q1	STxM > STxF	OA, Q3	IF > STxF	N.S	–
Count non-bout	Q2	IM > IF	OA, Q3	STxM > STxF	OA, Q1-4	IF > STxF	OA, Q1-4	IM > STxM
contraction freq	OA, Q1-4	IM > IF	Q1	STxM > STxF	OA, Q1-4	STxF > IF	N.S	–

*AUC* area under the curve, *Freq.* frequency, *OA* overall significant difference (regardless of quadrant), *N.S.* no significant differences, *Q* quadrant number from heatmap analysis (e.g. Fig. 4), *IM* intact male, *IF* intact female, *STxF* transected female, *STxM* transected male. > reflects direction of significant difference.

Between group differences had consistent patterns (Table 3) with intact males having greater mean amplitude, maximum amplitude, and mean range than intact females at 2 cm depth (both STIM ON and OFF), with intact females having greater mean AUC, mean duration, count (within bouts), and contraction frequency. Although at 10 cm intact males had significantly more activity across nearly all measures than intact females. Transected groups showed an effect of depth: transected females having significantly higher activity in all but 3 outcomes at 2 cm (STIM ON: mean AUC, mean duration; STIM OFF: mean duration) and transected males having significantly greater activity in all outcome measures at 10 cm. The pattern of elevated activity (both STIM ON and OFF) in these groups suggests a sex difference in colon function such that structures distant from the anus are more active in males whereas structures closer to the anus are more active in females, regardless of injury.

External anal sphincter (EAS) EMG recordings maintained consistent levels of baseline tonic activity, with periodic bursts of activity that were unrelated to the presence/absence of scES (Fig. 5D). High intensity stimulation (300–500 µA) enhanced EAS activity, with the greatest effect occurring in intact animals (Fig. 5C,D, green boxes). The percentage of animals showing EAS bursting in unison with EUS bursting was: 50% intact female (4/8), 33% intact male (3/9); and 0% transected males and females (Fig. 5C,D, red boxes), which likely reflects inhibition of bowel function during micturition^17^. This difference in the proportion of animals showing coincident EUS/EAS EMG activity between groups likely reflects impaired EUS activity post-transection.

### Visualized movement (VisMvt) effects from scES

In addition to scES-induced effects on bladder/bowel function, scES at L5-S1 also resulted in gross motor movements. scES applied over a large surface area was sufficient to activate motor pools of skeletal muscles in numerous regions (bilateral) that showed movement only during stimulation, including: axial muscles along spinal column, flanks (below ribs and above knee), hip, knee, ankle, toes, EAS, bulbo (males), scrotum (males), penis (males), and the base of the tail. At the higher intensities, the entire hindquarter would sometimes elevate. This elevation was considered a “complete response”, as once these movements occurred no other regions were recruited (up to 1 mA examined during pilot experiment, data not shown). Note that the pilot data determined the below and above VisMvt intensities utilized for mapping. Movement threshold, defined as the lowest stimulus intensity that caused a visible muscle movement in any of the above regions, were tested at the conclusion of mapping and was not statistically different between groups (SEM): Intact female = 321.25µA (51.2); transected female = 251.25 µA (25.9); intact male = 197.87µA (48.2); transected male = 233.125µA (52.99).

### Micro-leads array assessments

**Figure 7:**
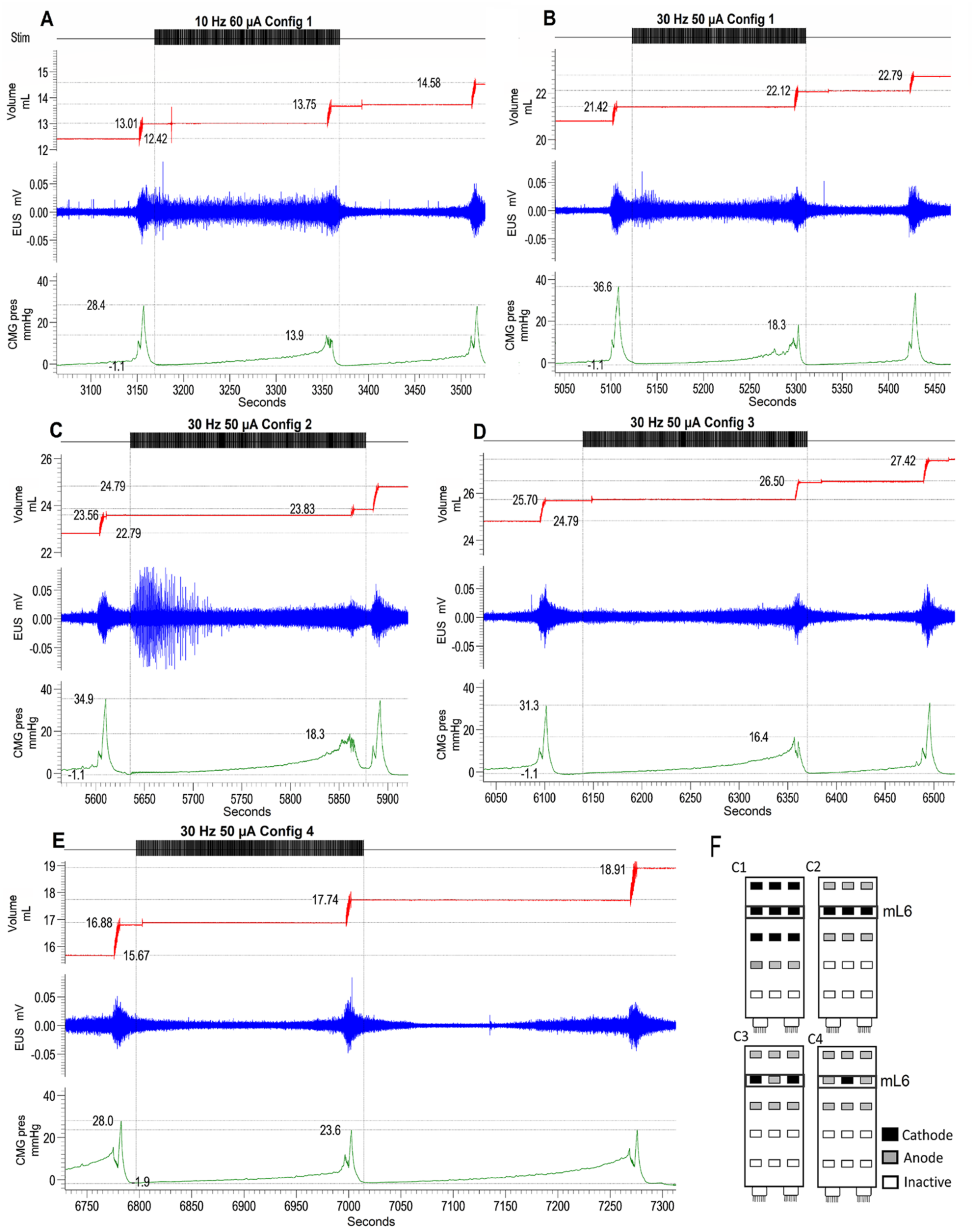
Comparison of the effect of different configuration with custom Micro-leads electrode array in Intact Females (n = 5). Initial testing of the electrode involved a replication of the stimulation surface area comparable to the modified Specify 5–6–5 Medtronic array (**A**,**B** examples; configuration 1 [**C1**] in **F**). Using *cathode stimulation* of L5-S1 with the Micro-Leads array achieved a similar suppression of voiding (reduced maximum pressure, lengthened inter-contractile interval, reduced void volume) in intact females at a much lower intensity of stimulation (50–60 µA vs. 300–500 µA) and without activation of gross motor movements **(A,B**). After replication, different anode and cathode configurations **(F**) were applied to determine site specificity of the effect on bladder function. It was found that a single strip of cathodes across medial L6 **(F,C2;** see in Fig. 1C) was sufficient to induce the changes in bladder function **(C**). Indeed, this configuration induced stronger EUS activation and a drastic reduction in maximum contraction pressure and expelled volume. The effect was lessened when cathode stimulation was applied to the lateral aspects of medial L6 **(F,C3; trace D**) and absent when only a single medial cathode was used **(F,C4; trace E**). Therefore, previous effects can be replicated by the custom electrode and furthermore, the effect can be isolated to a small region across the medial L6 level of the cord.

Subsequent testing on a group of 5 additional intact female rats using a custom-made 15-electrode array placed at L5-S1 initially involved replication of the stimulation surface area comparable to the modified Medtronic array (3 rows totaling 9 electrodes; see Figs. 1B and Fig. 7F—Configuration C1). The 9-electrode configuration achieved a similar disruption of voiding (reduced maximum pressure, lengthened inter-contractile interval, reduced void volume) at a much lower intensity of stimulation (50–80 µA vs. 300–500 µA) and without activation of gross motor movements (7A, B). Both 10 Hz and 30 Hz frequencies were tested in the initial replication (configuration C1); 30 Hz was used to test subsequent configuration changes. After replication, different anode and cathode configurations (Fig. 7F) were applied to determine site specificity of the effect on bladder function, by systematically reducing the number of electrodes with each fill-void ON/OFF sequence. It was found that a single strip of electrodes (C2 in Fig. 7F) mid-L6 (per electrode placement reconstructions, as shown in Fig. 1C) was sufficient to induce the changes in bladder function (7C). This configuration induced stronger EUS activation and a drastic reduction in maximum contraction pressure and expelled volume. The effect was lessened when scES was applied to only the dorsolateral aspects of L6 at the level of the dorsal root entry zone and absent when only a single dorsomedial cathode was used. Typical examples are provided in Fig. 7.

## Discussion

Initial pre-stimulation CMG recordings of the lower urinary tract (bladder pressure; EUS EMG) and bowel (distal colon pressure, rectal pressure, EAS EMG) in intact male and female rats showed typical response patterns in uninjured rodents. EAS EMG recordings maintained consistent levels of baseline tonic activity, with periodic bursting of activity. ^17^Responses of urinary and bowel systems to L5-S1 scES varied depending upon the parameters used, neurological intactness, sex, and specificity of the electrode, as discussed below. Taken together, this mapping data represents an important first step toward understanding both the location and stimulation parameters for specific response generation in bladder, sphincter (urethral and anal), and colorectal related functions.

### scES-induced lower urinary tract effects

The volume heat maps generated for varying frequencies and intensities of scES relative to equivalent OFF periods revealed two significant effects in spinally intact male and female rats. The first outcome occurred during fill-empty cycles with high frequency and above-VisMvt stimulation parameter combinations whereby the typical rise in bladder pressure upon reaching capacity did not generate a contraction. Instead, an extended duration and amplitude of tonic EUS activity (maintaining sphincter closure) occurred without expulsion of fluid. In addition, this scES-induced hold effect resulted in a state of overflow incontinence (OI)^18,19^ whereby the volume infused was leaking out in small droplets (as full capacity was reached). These low frequency leaks of fluid in combination with high CMG pressure ended immediately after stimulation offset with an afferent-driven reflex contraction of the bladder, EUS bursting, and expulsion of fluid. This storage-like effect is consistent with disruption of afferent driven bladder reflexes, and therefore subsequent bladder contraction, due to activation of the spinal descending inhibitory pathway from the pontine micturition center^20–23^. Occurrence of OI due to scES is consistent with previous work showing that afferent tibial nerve stimulation^24,25^, saphenous nerve stimulation^26,27^, and pudendal nerve stimulation^15,28^ can cause suppression of bladder activity and OI in rat models. This similarity is likely due to scES directly activating the spinal substrate/circuits that are also activated by afferent stimulation. Indeed, afferent projection sites are within the region of stimulation in this experiment (L3-L6 for tibial nerve stimulation). Note that due to substantial methodological differences between nerve stimulation studies and this one, any direct comparisons of the outcome data would be speculative.

The fill cycle was driven artificially for expedient mapping, at a supra-physiological rate, and we acknowledge that reaching a point where high CMG pressure occurs with prolonged holding could be detrimental if left over a long-extended time period. Thus, other lumbosacral cord locations are currently being mapped to identify a site that can either simultaneously reduce detrusor pressure or can extend the time between contractions without elevating pressure (initial data implicate T13-L1 spinal level; unpublished observations). Additionally, the increased activity in the EUS is likely a contributing factor, a finding consistent with scES directly activating Onuf’s nucleus and causing a guarding response^29^. Recent work in awake rats with lateral hemisection SCI shows that epidural stimulation has spatial specificity on bladder function, with L1 and L5-6 stimulation driving bladder contractions and sacral stimulation driving EUS function^30^. Additionally, it is also possible that current spread from the high intensity stimulation activated distal circuits. Therefore, previous data, in combination with these scES results, highlight the need for site-specific mapping of bladder circuits using high spatial resolution electrode arrays to tease apart spatial, frequency, and intensity specific effects.

A second major outcome, a significant elevation of void volume, occurred during fill-empty cycles with high frequency but below-VisMvt scES parameters. This finding is consistent with our previous scES human studies where similar patterns of 30 Hz and above frequencies at just below motor threshold intensities generated increases in reflexive bladder emptying in five male research participants, as measured in a controlled clinical laboratory setting during cystometry^8^. This effect is consistent with previous literature showing pudendal stimulation (between 20 and 50 Hz) can improve bladder contractions with minimal EUS involvement^15,28^. Therefore, it is possible that scES is activating circuits that cause pudendal outflow to aid bladder contractions and facilitate voiding.

In contrast with intact conditions, chronic spinal cord transection elicited a complete loss of EUS bursting activity during emptying. The lack of a bursting phenotype, which occurred regardless of whether scES was OFF or ON, is consistent with previous SCI data^31,32^. Although loss of bursting has been linked to urethane anesthesia^32^, its presence in intact rats suggests other factors are involved, such as loss due to a weakened and thus vulnerable state of the EUS muscle/neural control post-SCI. Note that a rise in activity still occurred during micturition, a pattern consistent with detrusor-sphincter dysynergia^20–23^ wherein the EUS contracts simultaneously with the detrusor and interferes with efficient bladder emptying. Thus, EUS activity post-transection was limited to a guarding reflex (increased tonicity) in response to increased luminal pressure during both filling and emptying.

In contrast to spinally intact rats, scES in transected animals at the same confirmed location generated an immediate increase in detrusor pressure, EUS EMG activity and expulsion of fluid, regardless of whether a typical micturition phenotype or one of overflow incontinence occurred. The pressure curves during these scES-induced events resemble the pressure dynamics and EUS EMG activities that occur during a detrusor contraction without stimulation in the intact state, suggesting that scES is causing a detrusor contraction to empty the bladder. The different outcomes for intact versus transected groups of rats support previous observations that chronic injury induces a reorganization of local urinary-related spinal circuits resulting from the loss of descending pathways between the pontine micturition center and the spinal micturition reflex circuitry^21–23,33^. Specifically, plasticity post-injury in numerous components of the micturition pathway including sodium channels on bladder afferents^34^, NGF activity^35,36^, and c-fiber afferent activity^37–41^ leads to an uninhibited reorganized reflex pathway that can be directly activated to trigger voiding via scES.

The triggering of strong bladder contractions would be beneficial to humans with SCI by possibly increasing void volume and thereby reducing residual volume. This neuromodulation approach could reduce reliance on catheterization. Increasing emptying efficiency is likely to reduce the rate of bladder infections^42,43^ and reduce inflammation^43^ from repeated catheterizations. Note that the current differences were found with a complete spinal transection. Whether or not the urinary system status reflects intact or complete transected conditions following incomplete injuries is currently under investigation using a clinically relevant contusion model.

### Sex differences impacting urinary tract function

One major finding was the sex difference in the proportion of animals showing an OI phenotype post-injury (33% females versus 100% males). Those animals without clear bladder contractions during fill/void cycles were deemed acontractile (no voiding; only presence of incontinent leaks). These animals were analyzed separately (Table 2). It was found that mean pressure, max pressure, and min pressure were significantly greater in transected females during below-VisMvt versus above-VisMvt stimulation intensities regardless of frequency. Fewer significant differences were found within transected males and may be due to the significantly greater AUC, mean pressure, maximum pressure, and minimum pressure of transected males than females in all but one instance. These quantitative differences may reflect an overly distended bladder in transected males that is not as able to contract regardless of stimulation. This is consistent with the transected male group having the lowest voided volume of all groups. Thus, with greater fluid content, the pressure and consequent measures would be greater.

Another major male–female difference relates to the presence of seminal plug material in the transected male urethra that accumulates after SCI, due to lowered thresholds for stimulation to elicit ejaculation, decreased force of expulsion, and dis-coordinated sphincter function leading to retrograde ejaculation^44–47^. It was also apparent during mapping that scES was causing further plug release, thus exasperating the passage and expulsion of urine. This difference is evident from the low void volume (light shade of blue in Fig. 3 maps) in transected versus intact males as well as transected females. Although presenting a methodological challenge, this model recapitulates the retrograde ejaculation phenomenon present in human males after SCI and therefore, could be used to investigate scES interventions to prevent this from occurring^46–48^. Additionally, the more specific Micro-Leads electrode array has not been tested in males and therefore it is not clear if plug release could be avoided with more localized targeted stimulation. Seminal plugs contain material released from the seminal vesicles^49^ which is also served by neural circuitries that includes the L5-S1 cord, the pelvic nerves as well as the hypogastric nerves^50–52^. Importantly, scES during mapping was applied over a large area without specific targeting, which could have activated ejaculatory related circuitry leading to plug material found during post-mortem examination.

### *scES-induced colorectal effects* overall

scES at the L5-S1 region caused reduced peristalsis activity. Sex differences were found between both intact and transected males and females. Specifically, intact females had greater values than males for mean AUC, mean duration, count bout, and contraction frequency (2 cm level), whereas intact males had greater mean amplitude, maximum amplitude, and mean range. This pattern was not present at the colonic level (10 cm), with intact males having greater activity in nearly all variables. These sex differences were not influenced by scES (same in both ON and OFF conditions). After transection, females had greater colorectal activity at 2 cm than males, whereas males had greater activity than females at 10 cm, regardless of stimulation application (Table 3, Intact and Transected columns).

The reduction in peristaltic activity was present in both intact and transected animals suggesting activation of a common pathway from the spinal cord to the rectum (2 cm from anal verge) and distal colon (10 cm from anal verge). In addition to a reduced number of contractions during stimulation, there was an elevated number upon scES offset. This rebound phenomenon (disinhibition) argues that the reduced activity during stimulation is inhibition of the basal activity of the organ. Unlike the urinary system, the similar response under spinally intact and chronic transection conditions indicates a lack of supra-spinal involvement as well as injury-induced plasticity of the neural circuitries, suggesting that scES is likely activating an underlying aspect of normal reflexive colonic function, possibly through spino-enteric interactions. In addition, the possibility exists that influencing bladder activity via L5-S1 scES may be indirectly activating circuitry that consequently inhibits bowel activity. For example, it is possible that high intensities are producing a spread of current to the T12-L1 sympathetic outflow^53^ and subsequent activation of sympathetic circuits are causing the decrease in activity. Additionally, the pelvic organs have a great deal of shared innervation, with overlapping central and peripheral substrates^53–55^, as pelvic organ crosstalk^56^ and coordinated activity exists under normal conditions.

### Electrode considerations

The custom fabricated Micro-Leads electrode array tested in intact females replicated the inhibition of micturition seen with the Medtronic electrode. Importantly, the effect was found at much lower stimulus intensities (50–90 µA vs. 300–500 µA) and occurred over a surface area confined to mid-L6, a region within the mapping area of the larger Medtronic array. Thus, the new multi-channel array, contoured for close contact over the entire medial–lateral surface of the cord, allowed for containment of the electrode within small boundaries, thereby limiting extent of current spread to adjacent areas. Additionally, because the stimulus intensity necessary for bladder contraction inhibition was so reduced, consequent gross motor movements associated with scES were rarely detected. The elimination of any body movements while preserving the effects on pelvic-visceral organs is crucial to the translational relevance of these data. It is imperative that any stimulation-induced intervention related to autonomic nervous system functions be below motor threshold in humans so that consequent motor activation does not limit the use of, impair the effectiveness of, or cause side effects during electrical stimulation. Furthermore, muscle contractions from stimulation could create a confounding variable during clinical trial studies. For example, abdominal contractions may indirectly cause a rise in detrusor pressure and/or the triggering of a void, which would be problematic for usage of these devices for bladder control in humans. Thus, having more available independently regulated contacts over a larger surface area will allow more precise control of multiple functional systems.

### Limitations and future directions

The use of an anesthetized model is problematic as even urethane, the most commonly used anesthesia for bladder function experiments, causes some depression of the urinary system. Therefore, it may be that the necessity of anesthesia has confounded the interpretation of some responses (i.e. lack of bladder contractions in transected males). Furthermore, the majority of the data presented here is using a very large, rather non-specific, electrode.

Now that the Micro-Leads array has been verified to not only reproduce the effect of the Medtronic electrode detailed in this paper but to do so at lower amplitudes, the next step is to conduct experiments without the need for anesthesia during testing. Additionally, it is now possible to design experiments that can apply highly site-specific scES to tease apart what nerves/pathways are responsible for the responses.

### Conclusions

Epidural spinal cord stimulation is a promising technology for numerous domains including reduction of chronic pain, motor control (stand, step, voluntary movements and over ground walking), and autonomic functions, including but not limited to the regulation of blood pressure and bladder management. Although there are neuromodulation devices that target specific aspects of lower urinary tract function such as continence^4^, the epidural approach holds promise as a singular device capable of influencing multiple body systems with user-controlled programs that can be selected (alone or in combination) at any given time of day or night depending on functional need (such as to stand, adjust blood pressure, prevent a bowel accident, or empty one’s bladder). The data presented here show the utility of epidural stimulation in a small animal model to understand basic functional control of the lower urinary tract and colorectal systems as well as how the effects on these systems change after chronic complete spinal cord injury. Importantly, it was shown that epidural stimulation of the L5-S1 cord can trigger an immediate void after spinal transection which could translate to a decreased reliance on catheterization in humans after SCI. Furthermore, scES influence on bowel function emphasizes the interconnected nature of the urinary and gastrointestinal systems as well as the ability of stimulation to influence the activity of two organ systems commonly impaired after injury. Therefore, these data represent an important step forward in understanding both the location and stimulation parameters for specific response generation in bladder and bowel function. Because intact and transected groups responded differently to scES, current studies are underway to determine the effects of scES in an incomplete model of SCI, as most injuries seen clinically are anatomically dis-complete, with a majority being functionally incomplete.

## Methods

All methods were carried out in accordance with ARRIVE guidelines.

### Animal groupings and spinal cord injury procedures

#### Animals

All animal procedures conformed to NIH guidelines and were reviewed and approved by the Institutional Animal Use and Care Committee at the University of Louisville, School of Medicine. A total of 20 female and 20 male Wistar rats were targeted for this mapping study and 5 female Wistar rats for Micro-Leads electrode testing. Half the animals (n = 10 females, n = 10 males) received a complete spinal cord transection at the T9 spinal level, whereas the intact groups (n = 10 females, n = 10 males) were not exposed to any surgical manipulation prior to terminal mapping procedures.

#### SCI

Spinal transections, as previously described^57,58^, were made under ketamine/xylazine anesthesia (80/10 mg/kg, intraperitoneally) with a microdissection scissor after exposure and removal of the T8 vertebra. Complete transection was visually confirmed and Gelfoam was packed into the vertebral space. The muscle layer was closed with 4–0 Ethicon absorbable suture whereas the cutaneous layer was closed with wound clips (Mikrotek, 9 mm autoclip).

### Spinal cord epidural stimulation (scES) electrode arrays

#### Modified medtronic array

A multi-electrode epidural stimulation array (Specify 5–6–5, Medtronic, Minneapolis, MN) was modified so that one lane of electrodes (5 contacts) was used to provide spinal cord stimulation during terminal testing procedures (Fig. 1B). This electrode has widespread use for modulating chronic pain^59,60^ and therefore the safety and efficacy of the device has been previously demonstrated. In recent years, the device has been repurposed and shown efficacy for step/stand training^61^, cardiovascular control^62^, and bladder function^8^ in SCI individuals. A total of two electrodes were placed on the epidural surface as a cathode (stimulation) and anode (ground) pair. Each electrode contact on this array is 2 mm W × 4 mm L with 4 mm between contacts. Therefore, each electrode covered the dorsomedial surface of the spinal cord up to the dorsal root entry zone on each side, as well as roughly 2–3 rostro-caudal levels of the cord. The large surface area covered by the electrode was chosen for this initial mapping study to minimize the possibility of false negatives in the region of interest. Thereafter, a custom scES 15-electrode array (Micro-Leads Inc., Somerville, MA, Fig. 1B) was designed and subsequently tested to further localize any stimulation effects within the region of interest.

#### Micro-leads array

The newly designed array is 11 mm long (9 mm contact length) by 2.5 mm wide and has five rows of 3 electrode contacts (0.5 mm × 0.5 mm LxW) for a total of 15 contacts (Fig. 1B). Three columns of contacts allow for stimulation of the medial (midline) and lateral (roughly at the dorsal root entry zone) aspects of the epidural surface. The five rows of contacts allow for configurations that can isolate the stimulation to approximately one rostro-caudal spinal level. By varying the anode and cathode positions, the scES site specificity can be investigated over an 18 mm^2^ area (with ~ 1 mm^2^ resolution). Wiring electrodes in series, rather than in parallel, ensured that all electrodes delivered the same amount of current when more than one was being used for stimulation. This was not necessary for the Medtronic electrode as there was only one cathode and one anode used. The Micro-Leads array is connected to a stimulator (Grass S88) via 32-pin connectors (Omnetics), an in-house fabricated connector made from epoxy and jumper cables, and breadboards wired in specific testing configurations (Fig. 7). Isolation units ensure constant current delivery from the S88 unit to the breadboards and then out to the epidural array.

### Terminal mapping study preparation

#### Anesthesia protocol

The intravenous (IV) route for urethane anesthesia was selected for this study, as intraperitoneal injection into the pelvic/abdominal space can have a suppressive effect on bladder function^63,64^ and it avoids variability in anesthetic depth with the subcutaneous route given the long experimental duration. The IV route also provides easy access (given the experimental setup) for supplemental anesthesia (urethane, 0.05 ml increments, 1.2 g/kg, 50% solution) as necessary.

#### Jugular catheter

Animals were initially anesthetized with Isoflurane (induction 5%, maintenance 2%) and placed in a supine position on a water-heated pad (Gaymar) to maintain body temperature. A sagittal skin incision (~ 1 cm) was made just lateral to the trachea and the tissues blunt dissected for access to the jugular vein and trachea. The jugular catheter was implanted by: isolating the vessel from connective tissue, tying off the rostral end with 4–0 silk suture, making a small incision (approx. 33% of the vessel width) in the vessel with microscissors, insertion of polyethylene tubing (PE-10, Intramedic, Clay Adams) into the vessel and securing the caudal end of the vessel around the catheter with silk suture, both the rostral and caudal ligatures are tied around the catheter to ensure orientation and structural strength. Anesthesia was transferred from isoflurane to IV urethane (1.2 g/kg) gradually by reducing the isoflurane percentage and slowly infusing urethane, over a 10–15-min period, and maintaining continuous surgical depth of anesthesia (measured by respiration rate and corneal response).

#### Tracheal catheter

After transitioning to urethane, a tracheal catheter was implanted to facilitate breathing over the long-term terminal study and provide a route to quickly remove respiratory secretions. The trachea was exposed by blunt dissection along the midline through the incision made previously for the jugular catheter implantation. A transverse incision was made between the tracheal cartilage and a Y-shaped tube was inserted and secured with silk suture (4–0).

#### Bladder catheter

Bladder catheters were implanted using previously published methods^65–67^. Briefly, catheter and wiring were implanted such that they were externalized via a scapular incision through a subcutaneous tunnel from the abdomen. After the abdominal incision was made, the fascia and abdominal muscles were opened via sharp and blunt dissection, respectively. The bladder dome was punctured with an 18-gauge needle and the catheter (PE-60 tubing with a heat-flared end) is inserted into the bladder dome and secured with a loop of silk suture (4–0).

#### EUS electrodes

The EUS is situated below the bladder neck, rostral to the pelvic symphysis and inguinal ligaments. Connective tissue and fascia were blunt dissected away to expose the striated muscle of the EUS. EUS electrode bundles are made from two lengths of thinner wire (A-M Systems, 0.002″ diameter, stainless steel) for placement into the EUS and one length of thicker wire (A-M Systems, 0.003″ wire) for a reference electrode. The thin wire is cut on a severe angle at one end to aid with insertion into the muscle. A loop is formed in the EUS electrodes by gently spinning them around a hook, made from a blunted needle, near the end (~ 2 cm) that will be inserted into the muscle. Using 6–0 suture (Ethicon), the wire loops were sutured to the inguinal ligament on each side. The stripped ends of the wires (~ 1–2 mm) were inserted into the EUS muscle bilaterally. Final closure of the abdominal muscles and skin was done with 4–0 suture. The ground wire was stripped, and a loop formed, as described above, and included in the abdominal stitches.

#### Laminectomy and electrode implantation

Epidural electrode implantation for the acute terminal experiments required a quadruple laminectomy to be performed, as previously described^65–67^. The very large size and moderate flexibility of the Medtronic electrode (one lane of 5–6–5 array) necessitates removal of numerous laminae to ensure contact of two electrode pads with the epidural surface, one for the anode and one for the cathode. Briefly, fine tip rongeurs were used to remove the lamina and expose the epidural surface after the skin, muscle, and fascia were incised. The electrode was implanted over the target segments (L5-S1) of the cord and secured by suturing the muscle layer together over the electrode. Because of the large electrode size, the mechanical hold of the muscle closure is all that is needed. The skin was closed with wound clips.

#### EAS electrodes

After epidural electrode placement, the rat was put into the prone position on a testing platform. The tail was clamped and elevated to allow observation of the anogenital area during testing. Fine wire hook electrodes were made by threading EMG wire (0.003″) into a 27-gauge needle, stripping a small segment (1–2 mm) near the needle, and bending the exposed end into a hook^61^. These electrodes were implanted bilaterally into the EAS muscle via an oblique angle beginning in the midline of the anus.

#### ARM probe insertion

Two pressure sensors (Millar, SPR-524, 3.5F) were inserted into the rectum (2 cm) and distal colon (10 cm)^68^. Any fecal pellets were first removed from the anus with two pairs of curved and blunt forceps. A length of large diameter polyethylene tubing (PE80) was inserted into the anus using non-spermicidal veterinary lubricant (Surgilube, HR Pharmaceuticals Inc, York, PA) and used as a speculum to insert the 10 cm probe. Once the tubing was at 10 cm depth, the sensor was threaded alongside the tubing to depth and secured with tape to the tail. The 2 cm probe was then inserted to depth and secured.

### Mapping study

#### Mapping procedure

After completion of the acute terminal preparation (see setup in Fig. 1A), the infusion pump was started and ran at a constant fill rate of 0.25 ml/min. Initial bladder pressure responses to the physiological saline infusion were observed during an acclamation period prior to baseline data collection. Because the bladder is quiescent under anesthesia and due to a 4–5-h preparation period, micturition cycle durations tend to be variable at the beginning of filling. Once the fill-void cycle had a consistent time between voids (similar inter-contraction interval, ICI), pre-mapping baseline data was collected for five fill-void cycles.

After the initial baseline testing, scES was applied (ON) followed by a non-stimulation period (OFF) at each of 30 parameter combinations. Based upon the animal literature for peripheral neuromodulation studies^15,17^ and our groups clinical studies^8^, five different frequencies representing a range of low and high frequencies were pre-selected (5 and 10 Hz; 30, 45, and 60 Hz, respectively). A list composed of six intensities (50, 75, 100, 150, 300, and 500 µA) were also pre-generated based upon a few pilot experiments (unpublished observations) examining VisMvt thresholds, so that both below and above VisMvt intensities (based upon any observed movements anywhere on the hindquarters) would be explored during bladder/bowel mapping. Other parameters used for all intensity/frequency combinations were as follows: 1 ms pulse duration, no delay, 1 train per second, and a 500 ms train duration. Two different parameter sequences were applied, both starting with 5 Hz 50 µA. Either the frequency was varied first (i.e. 5 Hz 50 µA, 10 Hz 50 µA, 30 Hz 50 µA, etc.…), or intensity was varied first (i.e. 5 Hz 50 µA, 5 Hz 75 µA, 5 Hz 100 µA, etc.…), until all 30 combinations were applied, with roughly half of the animals per group for each of the two sequences. After testing of all parameter combinations, two more post-mapping non-stimulation periods were collected.

A 2-min scES stimulus duration ON period and a 2-min OFF period was used to keep the experiments within a reasonable span of time based upon our previous neurophysiological studies and with regards to: the extensive prep-time, saline infusion acclimation period, pre-mapping baseline collection, 30-parameter testing time, and the post-mapping baseline period. However, if the ICI for a given animal was greater than 2 min without evidence of overflow incontinence (indicative of a full bladder), then a void-to-void stimulation sequence was used. In addition, if the scES induced a void (judged as not being reflexive based upon the pre-mapping baseline fill-void cycle pattern/duration), the stimulus was turned OFF for a 2-min period or until the next void occurrence if the ICI was greater than 2-min.

#### Micturition collections

The volume of fluid excreted during each period was measured by a balance (Ohaus, Scout) with a glass collecting beaker. Balance values were transmitted to the computer via a USB device interface cable (RS232) and software (Serial Port Data Collection, SPDC, Ohaus, V2.01; https://us.ohaus.com/en-us/support/software-and-drivers) such that values were put into an excel sheet on the computer whenever the mass was stable. Excel formulas were used to subtract the value of the current period from the previous period to calculate the volume per micturition. Micro-Leads pilot data (Fig. 7) was collected with a different apparatus for measuring the expelled fluid. A pressure transducer (Biopac Systems Inc., 250 g) modified with a collecting vial was used to collect expelled fluid and the signal was amplified (WPI Transbridge 4 M amplifier) and then exported directly into the data file (Spike 2, v8.15) via a CED Micro3 1401 unit. This setup allows for direct measurement of the expelled fluid in the same file, at the same time, as all of the other data traces.

#### Acquisition equipment

The bladder catheter was connected to a saline infusion pump and pressure sensor. All instrumentation traces were acquired using Cambridge Electronic Device (CED Micro3 1401) and Spike 2 (v8.15) software (http://ced.co.uk/products/spkovin). Bladder pressure (CMG) and electromyographic data (EUS, EAS) were amplified with a 4-channel pressure amplifier (WPI Transbridge 4 M amplifier) and a 4-channel differential AC amplifier (AM-Systems, model 1700), respectively. ARM probes (2 and 10 cm) (Millar SPF 524) were detected by the control unit (Millar) and recorded without further amplification.

#### Perfusion, tissue collection, and histology

After testing, motor threshold was assessed just prior to removal of instrumentation (i.e. ARM probes, scES electrode) and the electrode location was marked by putting a knot of suture in the dorsal musculature at the rostral and caudal aspects of each electrode pad. The knots of suture were then compared with the dorsal root entry zone (DREZ) at each level of the spinal cord (i.e. dashed line between R[rostral] and C[caudal] markings represents dorsal root entry zone, Fig. 1D) the next day to determine electrode placement. Instrumentation removal and electrode placement marking was followed by an anesthetic overdose (urethane, IV, 0.3 ml, 1.2 g/kg 50% solution) and trans-cardiac perfusion with heparinized phosphate-buffered saline (300 ml, 0.9% NaCl) followed by paraformaldehyde (4%, 300 ml). Spinal cord tissue was collected after fixation for verification of complete transection. Fixed tissue was post-fixed in 4% paraformaldehyde for at least 1 day then moved into a sucrose solution (30%), prior to sectioning. Sagittal Sects. (35 µm thickness, Cryostat) were thaw-mounted, stained with Luxol fast blue (white matter) and transection confirmed, per established protocols^59–62.^.

### Data quantification and analyses

#### Outcome trace quantification

Custom software was developed in Matlab to quantify and collect the data from raw signal traces (Fig. 1E–G). The types of data collected from the traces included the following 15 parameters: CMG trace (micturition volume [cc], bladder pressure [mmHg], maximum contractile pressure [mmHg], ICI [sec], AUC of contraction [mmHg.sec], duration of contraction [sec]); EUS- EMG (total duration of activity [sec], duration of tonic activity [sec], duration of phasic/bursting activity, maximum amplitude [mmHg]); and ARM traces (number of contractions within bouts, number of non-bout contractions, average amplitude [mmHg], maximum amplitude [mmHg], frequency of contraction). Additionally, any observed motor movements during stimulation were recorded manually during testing.

#### Heat map creation

To generate a continuous color-coded map (heat map) for a measured parameter (e.g., inter-contractile time), a two-dimensional (2D) matrix with all possible frequency and intensity pairs is constructed (i.e., size is 6 × 5). Each matrix value, at a given frequency/ intensity pair, represents the mean value of the measured parameter from all animals of a given group (i.e., intact females, intact males, transected females, or transected males). Once all possible frequency/intensity pair mean values are calculated, a finer 2D mesh is generated and a spline interpolation is applied to create a finer matrix. Finally, color-coded maps are constructed for the estimated parameters for visualization and the same color scale (for each parameter) is used for the comparison between animal groups and stimulation status (i.e., ON and OFF).

#### Statistical analysis:

Data was analyzed using mixed linear models regressing the outcomes on sex, injury (no = Intact, yes = transected), stimulation (yes/no), stimulation frequency, and intensity as well as their interactions. To capture within entity variability, a random intercept was added for each rat. Random slopes for stimulation, stimulation frequency, and stimulation intensity were also included to capture individual trends. Outcomes were presented by least square means and standard error from these models. Differences studied were obtained by building linear contrasts on the Sex*Stimulation*Injury*Frequency*Intensity* interaction. The significance level was set to 0.05 and all tests were 2-sided. Statistical analyses were performed in SAS 9.4 (SAS Inc, Cary, NC).

## Supplementary Information

Supplementary Information

## Data Availability

As part of the NIH SPARC Materials Sharing policy, the curated datasets generated and/or analyzed for the current study are available at 10.26275/u17s-hcn0.
