# Supplementary material for: Bladder and bowel responses to lumbosacral epidural stimulation in uninjured and transected anesthetized rats
Source: Sci Rep. 2021 Feb 8;11:3268. doi: 10.1038/s41598-021-81822-3 (PMC7870824; doi:10.1038/s41598-021-81822-3)
Supplement: Supplementary file 1 — Supplementary Information [file 41598_2021_81822_MOESM1_ESM.pdf]

Supplement for:

Bladder and bowel responses to lumbosacral epidural stimulation in uninjured and transected anesthetized rats.

Robert F. Hoey, Daniel Medina-Aguiñaga, Fahmi Khalifa, Beatrice Ugiliweneza, Sharon Zdunowski, Jason Fell, Ahmed Naglah, Ayman S. El-Baz, April N. Herrity, Susan J. Harkema, and Charles H. Hubscher*.

Supplemental Table 1a - Bladder Outcomes Between Group Differences (STIM ON)				
	Female	Male	Intact	Transected
Volume (cc)	OA: IF> STxF, p=0.0032<br>Q1: IF > STxF, p=0.0078<br>Q2: IF > STxF, p<0.0001<br>Q3: STxF > IF, p<0.0001	OA: IM > STxM, p<0.0001<br>Q1-4: IM > STxM, p<0.0001	OA: IF > IM, p=0.0433<br>Q2: IF > IM, p=0.0095	OA: STxF > STxM, p<0.0001<br>Q1: STxF > STxM, p=0.0012<br>Q2: STxF > STxM, p=0.0007<br>Q3: STxF > STxM, p<0.0001<br>Q4: STxF > STxM, p=0.0013
Intercontractile Interval (sec)	Q3: IF > STxF, p=0.0028	N.A.	N.S.	N.A.
Maximum Contractile Pressure (mmHg)	Q3: IF > STxF, p=0.0071	N.A.	OA: IM > IF, p=0.0104<br>Q4: IM > IF, p=0.0307	N.A.
Cystometrogram AUC (mmHg.sec)	Q3: IF > STxF, p=0.0068	N.A.	N.S.	OA: STxM > STxF, p<0.0001<br>Q1-4: STxM > STxF, p<0.0001
Non-voiding Contractions	N.S.	N.A.	N.A.	N.A.
EMG Total Activity Time (sec)	OA: STxF > IF, p=0.0474<br>Q1: STxF > IF, p=0.0186<br>Q2: STxF > IF, p=0.0001	N.A.	OA: IF > IM, p<0.0001<br>Q1-3: IF > IM, p<0.0001<br>Q4: IF > IM, p=0.0002	N.A.
EMG Mean Bursting Time (sec)	N.A.	N.A.	OA: IM > IF, p<0.0001<br>Q1, Q2: IM > IF, p<0.0001<br>Q3: IM > IF, p=0.0012	N.A.
EMG Burst Freq (Hz)	N.A.	N.A.	OA: IF> IM, p=0.0006<br>Q1: IF > IM, p=0.003<br>Q2: IF > IM, p<0.0001	N.A.
EMG Bursting On:Off ratio	N.A.	N.A.	Q2: IF > IM, p=0.014	N.A.
EUS-EMG Bursting Time (sec)	N.A.	N.A.	OA: IM > IF, p<0.0001<br>Q1,2,4: IM > IF, p<0.0001<br>Q3: IM > IF, p=0.0027	N.A.

Supplemental Table 1b - Bladder Outcomes Between Group Differences (STIM OFF)				
	Female	Male	Intact	Transected
Volume (cc)	OA: IF > STxF, p<0.0001<br>Q1: IF > STxF, p=0.0078<br>Q2: IF > STxF, p=0.0028<br>Q3: IF > STxF, p=0.0014<br>Q4: IF > STxF, p=0.0338	OA: IM > STxM, p<0.0001<br>Q1-3: IM > STxM, p<0.0001<br>Q4: IM > STxM, p=0.0003	N.S.	OA: STxF > STxM, p<0.0001<br>Q1: STxF > STxM, p=0.0009<br>Q2: STxF > STxM, p<0.0001<br>Q3: STxF > STxM, p<0.0001<br>Q4: STxF > STxM, p=0.0046
Intercontractile Interval (sec)	N.S.	N.A.	N.S.	N.A.
Maximum Contractile Pressure (mmHg)	N.S.	N.A.	N.S.	N.A.
Cystometrogram AUC (mmHg.sec)	N.S.	N.A.	N.S.	OA: STxM > STxF, p<0.0001<br>Q1-4: STxM > STxF, p<0.0001
Non-voiding Contractions	Q3: STxF > IF, p=0.0015	N.A.	N.A.	N.A.
EMG Total Activity Time (sec)	OA: STxF > IF, p<0.0001<br>Q1-2: STxF > IF, p<0.0001	N.A.	OA: IF > IM, p<0.0001<br>Q1, Q2: IF > IM, p<0.0001<br>Q3: IF > IM, p=0.0003<br>Q4: IF > IM, p=0.0023	N.A.
EMG Mean Bursting Time (sec)	N.A.	N.A.	OA: IM > IF, p<0.0001<br>Q1: IM > IF, p=0.0121<br>Q2, Q3: IM > IF, p<0.0001	N.A.
EMG Burst Freq (Hz)	N.A.	N.A.	OA, Q1-3: IF > IM, p<0.0001<br>Q4: IF > IM, p=0.0027	N.A.
EMG Bursting On:Off ratio	N.A.	N.A.	OA: IF > IM, p=0.0012<br>Q1: IF > IM, p=0.0264<br>Q3: IF > IM, p=0.0323<br>Q4: IF > IM, p=0.0077	N.A.
EUS-EMG Bursting Time (sec)	N.A.	N.A.	OA: IM > IF, p<0.0001<br>Q1-4: IM > IF, p<0.0001	N.A.

Supplemental Table 2a - Bladder Outcomes Within Group Differences (STIM ON)				
	Intact Female	Transected Female	Intact Male	Transected Male
Volume (cc)	Q2 >Q1, p=0.0185<br>Q2 > Q3, p=0.0098<br>Q2 On > Off, p=0.0212	Q3 > Q1, p<0.0001<br>Q3 > Q4, p<0.0001<br>Q3 > Q2, p<0.0001<br>OA: On > Off, p=0.0142<br>Q3 On > Off, p<0.0001	N.S.	N.S.
Intercontractile Interval (sec)	Q3 > Q1, p=0.0008<br>Q3 > Q2, p=0.0003<br>Q3 > Q4, p=0.002<br>Q3: On > Off, p=0.0003	N.S.	N.S.	N.A.
Maximum Contractile Pressure (mmHg)	Q1 > Q4, p=0.0338	Q1 > Q3, p<0.0001<br>Q2 > Q3, p=0.0003<br>Q4 > Q3, p=0.028<br>Q3: Off > On, p=0.0012	N.S.	N.A.
Cystometrogram AUC (mmHg.sec)	Q3 > Q1, p=0.0023<br>Q3 > Q2, p=0.0007<br>Q3 > Q4, p=0.0047<br>Q3: On > Off, p=0.0005	N.S.	N.S.	N.S.<br>OA: Off > On, p=0.0293<br>Q3: Off > On, p=0.0353<br>Q4: Off > On, p<0.0001
Non-voiding Contractions	N.S.	Q1 > Q3, p=0.0412<br>Q3: Off > On, p<0.0001<br>Q4: Off > On, p=0.0464	N.A.	N.A.
EMG Total Activity Time (sec)	N.S.	Q1 > Q3, p=0.0041<br>Q1 > Q4, p=0.016<br>Q2 > Q1, p=0.0326<br>Q2 > Q3, p<0.0001<br>Q2 > Q4, p=0.0002<br>OA: Off > On, p<0.0001<br>Q1: Off > On, p=0.0003<br>Q3: Off > On, p=0.0125	N.S.	N.A.
EMG Mean Bursting Time (sec)	Q3 > Q4, p=0.0061<br>Q4: Off > On, p=0.0006	N.A.	Q1 > Q4, p=0.0172<br>Q2 > Q4, p=0.0006<br>Q3 > Q4, p=0.0011	N.A.
EMG Burst Freq (Hz)	Q1 > Q3, p<0.0001<br>Q1 > Q4, p=0.0004<br>Q2 > Q3, p<0.0001<br>Q2 > Q4, p=0.0121<br>OA: Off > On, p=0.0035<br>Q3: Off > On, p=0.0003<br>Q4: Off > On, p=0.0175	N.A.	N.S.	N.A.
EMG Bursting On:Off ratio	Q1 > Q3, p=0.0006<br>Q1 > Q4, p=0.0043<br>Q2 > Q3, p=0.0003<br>Q2 > Q4, p=0.0042<br>OA: Off > On, p=0.0016<br>Q3: Off > On, p=0.0039<br>Q4: Off > On, p=0.0009	N.A.	Q1 > Q2, p=0.0265	N.A.
EUS-EMG Bursting Time (sec)	Q3 > Q1, p<0.0001<br>Q3 > Q2, p<0.0001<br>Q3 > Q4, p=0.0306<br>OA: On > Off, p=0.0118<br>Q3: On > Off, p=0.0006<br>Q4: On > Off, p=0.0485	N.A.	Q2 > Q1, p=0.0222<br>Q4 > Q1, p=0.0246	N.A.

Supplemental Table 2b - Bladder Outcomes Within Group Differences (STIM OFF)				
	Intact Female	Transected Female	Intact Male	Transected Male
Volume (cc)	N.S.	N.S.	N.S.	N.S.
Intercontractile Interval (secl)	N.S	N.S	N.S.	N.A.
Maximum Contractile Pressure (mmHg)	N.S.	N.S.	N.S.	N.A.
Cystometrogram AUC (mmHg.sec)	N.S.	N.S.	N.S.	Q4 > Q1, p<0.0001<br>Q4 > Q2, p<0.0001<br>Q4 > Q3, p<0.0001
Non-voiding Contractions	N.S.	Q3 > Q1, p<0.0001<br>Q3 > Q2, p<0.0001<br>Q3 > Q4, p=0.0077	N.S.	N.A.
EMG Total Activity Time (sec)	N.S.	Q1 > Q3, p=0.0019<br>Q1 > Q4, p=0.0026<br>Q2 > Q3, p=0.0104<br>Q2 > Q4, p=0.017	N.S.	N.A.
EMG Mean Bursting Time (sec)	Q4 > Q2, p=0.0037<br>Q4 > Q3, p=0.0234	N.A.	Q3 > Q1, p=0.0092<br>Q3 > Q4, p=0.0058	N.A.
EMG Burst Freq (Hz)	N.S.	N.A.	Q4 > Q3, p<0.0001	N.A.
EMG Bursting On:Off ratio	N.S.	N.A.	N.S.	N.A.
EUS-EMG Bursting Time (sec)	N.S.	N.A.	Q3 > Q1, p=0.0331	N.A.

Supplemental Table 3a – 2 cm Bowel Outcomes Between Group Differences (STIM ON)				
	Female	Male	Intact	Transected
Mean Amplitude (mmHg)	OA: STxF > IF, p<0.0001<br>Q1,2,4: STxF > IF, p<0.0001<br>Q3: STxF > IF, p=0.0001	OA: IM > STxM, p=0.001<br>Q3: IM > STxM, p<0.0001	OA: IM > IF, p<0.0001<br>Q1-4: IM > IF, p<0.0001	OA: STxF > STxM, p=0.0002<br>Q1: STxF > STxM, p=0.002<br>Q2: STxF > STxM, p=0.0096
Maximum Amplitude (mmHg)	OA: STxF > IF, p<0.0001<br>Q1: STxF > IF, p<0.0001<br>Q2: STxF > IF , p=0.0002<br>Q4: STxF > IF, p=0.0026	OA: IM > STxM , p=0.0001<br>Q2: IM > STxM , p=0.0303<br>Q3: IM > STxM, p<0.0001<br>Q4: IM > STxM, p=0.0256	OA: IM > IF, p<0.0001<br>Q2: IM > IF , p=0.0067<br>Q3: IM > IF, p<0.0001<br>Q4: IM > IF, p=0.0021	OA: STxF > STxM, p<0.0001<br>Q1: STxF > STxM, p=0.0004<br>Q2: STxF > STxM, p=0.0012<br>Q4: STxF > STxM, p=0.0114
Mean AUC (mmHg.sec)	Q1: STxF > IF, p=0.0384	OA: STxM > IM, p<0.0001<br>Q1-2: STxM > IM, p<0.0001<br>Q3: STxM > IM, p=0.0025<br>Q4: STxM > IM, p=0.0009	OA: IF > IM, p=0.0188<br>Q1: IF > IM, p<0.0001<br>Q2: IF > IM, p=0.0345<br>Q3: IM > IF, p=0.0425	OA: STxM > STxF, p<0.0001<br>Q1: STxM > STxF, p=0.0012<br>Q2: STxM > STxF, p=0.0196<br>Q3: STxM > STxF, p=0.0005
Mean Duration (sec)	OA: IF > STxF, p<0.0001<br>Q1: IF > STxF, p<0.0001<br>Q2: IF > STxF, p=0.0021<br>Q4: IF > STxF, p=0.0223	OA: STxM > IM, p<0.0001<br>Q1, 2: STxM > IM, p<0.0001<br>Q3: STxM > IM, p=0.043<br>Q4: STxM > IM, p=0.0195	OA: IF > IM, p<0.0001<br>Q1,2: IF > IM, p<0.0001<br>Q4: IF > IM, p=0.0269	OA: STxM > STxF, p<0.0001<br>Q1,2: STxM > STxF, p<0.0001<br>Q3: STxM > STxF, p=0.0047<br>Q4: STxM > STxF, p=0.0102
Mean Range	OA: STxF > IF, p<0.0001<br>Q1,2: STxF > IF, p<0.0001<br>Q3: STxF > IF, p=0.0284<br>Q4: STxF > IF, p=0.0004	Q3: IM > STxM, p=0.0031	OA: IM > IF, p<0.0001<br>Q2: IM > IF, p=0.0007<br>Q3,4: IM > IF, p<0.0001	OA: STxF > STxM, p=0.0005<br>Q1: STxF > STxM, p=0.0019<br>Q2: STxF > STxM, p=0.0039
Contraction Count (within bouts)	OA: IF > STxF, p<0.0001<br>Q2: IF > STxF, p<0.0001<br>Q3: IF > STxF, p=0.0001	OA: IM > STxM, p<0.0001<br>Q1: IM > STxM, p=0.001<br>Q2,3: IM > STxM, p<0.0001<br>Q4: IM > STxM, p=0.0012	Q2: IF > IM, p=0.0127	OA: STxF > STxM, p=0.037
Contraction Count (non-bout)	N.S.	Q2: STxM > IM, p=0.0445	N.S.	N.S.
Contraction Frequency	OA: IF > STxF, p=0.0454<br>Q2: IF > STxF, p=0.0287	OA: IM > STxM, p<0.0001<br>Q1,3,4: IM > STxM, p≤0.0457<br>Q2: IM > STxM, p=0.0001	OA: IF > IM, p=0.0096<br>Q2: IF > IM, p=0.0034	OA: STxF > STxM, p<0.0001<br>Q1: STxF > STxM, p=0.0002<br>Q2: STxF > STxM, p=0.0014<br>Q3: STxF > STxM, p=0.01<br>Q4: STxF > STxM, p<0.0001

Supplemental Table 3b – 2 cm Bowel Outcomes Between Group Differences (STIM OFF)				
	Female	Male	Intact	Transected
Mean Amplitude (mmHg)	OA: STxF > IF, p<0.0001<br>Q1-3: STxF > IF, p<0.0001<br>Q4: STxF > IF, p=0.0029	Q3: IM > STxM , p=0.0424	OA: IM > IF, p<0.0001<br>Q1-4: IM > IF, p<0.0001	OA: STxF > STxM, p<0.0001<br>Q1: STxF > STxM, p<0.0001<br>Q2: STxF > STxM, p=0.0005<br>Q3: STxF > STxM, p=0.0027
Maximum Amplitude (mmHg)	OA: STxF > IF, p<0.0001<br>Q1-3: STxF > IF, p<0.0001	OA: IM > STxM, p=0.0207	OA: IM > IF, p=0.002<br>Q2: IM > IF, p=0.0282	OA: STxF > STxM, p<0.0001<br>Q1-3: STxF > STxM, p<0.0001
Mean AUC (mmHg.sec)	OA: STxF > IF, p<0.0001<br>Q1: STxF > IF, p=0.0013<br>Q2: STxF > IF, p=0.0108<br>Q3: STxF > IF, p=0.0089	OA: STxM > IM, p<0.0001<br>Q1-3: STxM > IM, p<0.0001<br>Q4: STxM > IM, p=0.0002	OA: IF > IM, p=0.0002<br>Q1: IF > IM, p=0.0047<br>Q2: IF > IM, p=0.0044<br>Q4: IF > IM, p=0.046	N.S.
Mean Duration (sec)	OA: IF > STxF, p=0.0004<br>Q1: IF > STxF, p=0.0014<br>Q2: IF > STxF, p=0.0024	OA: STxM > IM, p<0.0001<br>Q1-4: STxM > IM, p<0.0001	OA: IF > IM, p<0.0001<br>Q1,2: IF > IM, p<0.0001<br>Q3: IF > IM, p=0.0203<br>Q4: IF > IM, p=0.001	OA: STxM > STxF, p<0.0001<br>Q1-4: STxM > STxF, p<0.0001
Mean Range	OA: STxF > IF, p<0.0001<br>Q1-3: STxF > IF, p<0.0001<br>Q4: STxF > IF, p=0.039	N.S.	OA: IM > IF, p<0.0001<br>Q1: IM > IF, p=0.0327<br>Q2: IM > IF, p=0.0076<br>Q3: IM > IF, p=0.0001<br>Q4: IM > IF, p=0.0232	OA: STxF > STxM, p<0.0001<br>Q1,2: STxF > STxM, p<0.0001<br>Q3: STxF > STxM, p=0.0037
Contraction Count (within bouts)	OA: IF > STxF, p=0.0267<br>Q3: IF > STxF, p=0.0003	OA: IM > STxM,p<0.0001<br>Q1: IM > STxM, p=0.0024<br>Q2: IM > STxM, p<0.0001<br>Q3: IM > STxM, p=0.0024	OA: IF > IM, p<0.0001<br>Q1: IF > IM, p=0.0076<br>Q2: IF > IM, p=0.0269<br>Q3: IF > IM, p<0.0001	OA: STxF > STxM, p<0.0001<br>Q1,2: STxF > STxM, p<0.0001<br>Q3: STxF > STxM, p=0.006
Contraction Count (non-bout)	N.S.	N.S.	OA: IF > IM, p=0.0014<br>Q3: IF > IM, p<0.0001<br>Q4: IF > IM, p=0.013	N.S.
Contraction Frequency	N.S.	OA: IM > STxM, p=0.0014<br>Q1: IM > STxM, p=0.0037	OA: IF > IM, p=0.0006<br>Q1: IF > IM, p=0.0184<br>Q2: IF > IM, p=0.0411<br>Q3: IF > IM, p=0.0018	OA: STxF > STxM, p<0.0001<br>Q1,2: STxF > STxM, p<0.0001<br>Q3: STxF > STxM, p=0.0138

Supplemental Table 4a – 2 cm Bowel Outcomes Within Group Differences (STIM ON)				
	Intact Female	Transected Female	Intact Male	Transected Male
Mean Amplitude (mmHg)	N.S.	OA: Off > On, p=0.0363<br>Q3: Off > On, p=0.0051	Q2 > Q1, p=0.0046<br>Q3 > Q1, p<0.0001<br>Q4 > Q1, p=0.0025<br>Q3 > Q2, p=0.0001<br>Q3 > Q4, p=0.0188	N.S.
Maximum Amplitude (mmHg)	N.S.	OA: Off > On, p=0.0005<br>Q2: Off > On, p=0.0029<br>Q3: Off > On, p=0.0001	Q2 > Q1, p=0.004<br>Q3 > Q1, p=<0.0001<br>Q4 > Q1, p=0.0106<br>Q3 > Q2, p=0.0009<br>Q3 > Q4, p=0.0137<br>Q3: On > Off, p=0.0465	N.S.
Mean AUC (mmHg.sec)	N.S.	OA: Off > On, p=0.0022<br>Q3: Off > On, p=0.0022	Q2 > Q1, p=0.0004<br>Q3 > Q1, p<0.0001<br>Q3 > Q2, p=0.0171<br>Q3 > Q4, p=0.0076	Q1: On > Off, p=0.046
Mean Duration (sec)	Q1 > Q2, p=0.0266<br>Q1 > Q3, p=0.0022	OA: Off > On, p=0.0246<br>Q3: Off > On, p=0.0088	N.S.	Q1 > Q3, p=0.0214<br>Q2 > Q3, p=0.0022<br>Q2 > Q4, p=0.0183<br>OA: Off > On, p<0.0001<br>Q3: Off > On, p<0.0001<br>Q4: Off > On, p=0.0003
Mean Range	Q2 > Q1, p=0.0416<br>Q2 > Q4, p=0.0302	OA: Off > On, p=0.0242<br>Q3: Off > On, p=0.0041	Q2 > Q1, p=0.0017<br>Q3 > Q1, p<0.0001<br>Q4 > Q1, p=0.0072<br>Q3 > Q2, p=0.0002<br>Q3 > Q4, p=0.0041	N.S.
Contraction Count (within bouts)	Q2 > Q1, p=0.0067<br>Q2 > Q4, p=0.0229<br>OA: Off > On, p=0.0103<br>Q3: Off > On, p<0.0001	OA: Off > On, p=0.0004<br>Q2: Off > On, p=0.0054<br>Q3: Off > On, p<0.0001	Q3 > Q1, p=0.0168	Q4: Off > On, p=0.0449
Contraction Count (non-bout)	Q3 > Q1, p=0.0482<br>Q4 > Q1, p=0.0293<br>Q3 > Q2, p=0.0286<br>Q4 > Q2, p=0.0488<br>OA: Off > On, p=0.0026<br>Q3: Off > On, p<0.0001	OA: Off > On, o=0.0123<br>Q3: Off > On, p=0.0037	N.S.	Q3 > Q1, p=0.0458
Contraction Frequency	Q2 > Q1, p=0.0109<br>Q3 > Q1, p=0.0039	OA: Off > On, p=0.0107<br>Q1: Off > On, p=0.0044<br>Q2: Off > On, p=0.0029	Q3 > Q1, p=0.0011<br>Q3 > Q2, p=0.0046<br>Q3 > Q4, p=0.0279	Q2 > Q1, p=0.0178<br>Q2 > Q4, p=0.0238<br>Q3 > Q4, p=0.0417<br>OA: Off > On, p=0.0101<br>Q4: Off > On, p=0.0051

Supplemental Table 4b – 2 cm Bowel Outcomes Within Group Differences (STIM OFF)				
	Intact Female	Transected Female	Intact Male	Transected Male
Mean Amplitude (mmHg)	N.S.	Q3 > Q4, p=0.0157	Q3 > Q1, p<0.0001<br>Q4 > Q1, p=0.0095<br>Q3 > Q2, p=0.0041	N.S.
Maximum Amplitude (mmHg)	Q3 > Q1, p=0.0173<br>Q4 > Q1, p=0.0314	Q2 > Q4, p=0.0288<br>Q3 > Q4, p=0.0095	Q3 > Q1, p=0.0049<br>Q4 > Q1: p=0.0164	N.S.
Mean AUC (mmHg.sec)	N.S.	N.S.	Q3 > Q1, p=0.0175	N.S.
Mean Duration (sec)	N.S.	N.S.	N.S.	Q3 > Q1, p=0.0024<br>Q3 > Q2, p=0.0002<br>Q4 > Q2, p=0.0427
Mean Range	N.S.	Q3 > Q4, p=0.0316	N.S.	N.S.
Contraction Count (within bouts)	Q3 > Q1, p<0.0001<br>Q3 > Q2, p<0.0001<br>Q3 > Q4, p<0.0001	N.S.	Q2 > Q1, p=0.0201<br>Q3 > Q1, p=0.0012<br>Q4 > Q1, p=0.0055	N.S.
Contraction Count (non-bout)	Q3 > Q1, p<0.0001<br>Q4 > Q1, p<0.0001<br>Q3 > Q2, p<0.0001<br>Q4 > Q2, p=0.0039<br>Q3 > Q4, p=0.0168	Q3 > Q1, p=0.0038<br>Q3 > Q2, p=0.0282<br>Q3 > Q4, p=0.0268	N.S.	Q3 > Q1, p=0.026
Contraction Frequency	Q3 > Q1, p=0.0012<br>Q3 > Q2, p=0.0105<br>Q3 > Q4, p=0.0041	Q2 > Q4, p=0.0148<br>Q3 > Q4, p=0.0222	Q2 > Q1, p=0.0299<br>Q3 > Q1, p=0.0224<br>Q4 > Q1, p=0.027	Q2 > Q1, p=0.006<br>Q3 > Q1, p=0.0482

Supplemental Table 5a – 10 cm Bowel Outcomes Between Group Differences (STIM ON)				
	Female	Male	Intact	Transected
Mean Amplitude (mmHg)	OA: STxF > IF, p=0.0064<br>Q1: STxF > IF, p<0.0001	OA: IM > STxM, p=0.0097<br>Q1: IM > STxM, p=0.0198<br>Q2: IM > STxM, p=0.0173	OA: IM > IF, p<0.0001<br>Q1: IM > IF, p<0.0001<br>Q3: IM > IF, p=0.0009<br>Q4: IM > IF, p=0.0349	N.S.
Maximum Amplitude (mmHg)	Q1: STxF > IF, p=0.0001	OA: IM > STxM, p=0.0035<br>Q1: IM > STxM, p=0.0052<br>Q2: IM > StxM, p=0.0044	OA: IM > IF, p<0.0001<br>Q1: IM > IF, p<0.0001<br>Q2: IM > IF, p=0.0054<br>Q4: IM > IF, p=0.026	N.S.
Mean AUC (mmHg.sec)	OA: STxF > IF, p=0.0007<br>Q1: STxF > IF, p<0.0001<br>Q2: STxF > IF, p=0.0218	OA: IM > STxM, p=0.0075<br>Q3: IM > STxM, p=0.0402<br>Q4: IM > STxM, p=0.0354	OA: IM > IF, p<0.0001<br>Q1-4: IM > IF, p<0.0001	OA: STxM > STxF, p<0.0001<br>Q1,2: STxM > STxF, p<0.0001
Mean Duration (sec)	OA: IF > STxF, p=0.027<br>Q1: IF > STxF, p=0.0079	Q1: IM > STxM, p=0.0071<br>Q3: STxM > IM, p=0.0005<br>Q4: IM > STxM, p=0.0408	OA: IM > IF, p<0.0001<br>Q1-4: IM > IF, p<0.0001	OA: STxM > STxF, p<0.0001<br>Q1,2: STxM > STxF, p<0.0001<br>Q3: STxM > STxF, p=0.0473<br>Q4: STxM > STxF, p=0.0005
Mean Range	OA: STxF > IF, p=0.0029<br>Q1: STxF > IF, p<0.0001	N.S.	OA: IM > IF, p<0.0001<br>Q1: IM > IF, p<0.0001<br>Q2: IM > IF, p=0.0001<br>Q4: IM > IF, p=0.0135	N.S.
Contraction Count (within bouts)	N.S.	N.S.	Q1: IM > IF, p=0.0017	OA: STxM > STxF, p=0.035<br>Q1: STxM > STxF, p=0.0126
Contraction Count (non-bout)	OA: IF > STxF, p<0.0001<br>Q1: IF > STxF, p=0.0019<br>Q2: IF > STxF, p=0.0029<br>Q3: IF > STxF, p<0.0001<br>Q4: IF > STxF, p=0.0026	OA: IM > STxM, p<0.0001<br>Q1: IM > STxM , p=0.0069<br>Q2: IM > STxM, p<0.0001<br>Q4: IM > STxM, p=0.002	OA: IM > IF, p=0.0061<br>Q2: IM > IF, p=0.0003	OA: STxM > STxF, p=0.0016<br>Q2: STxM > STxF, p=0.0323<br>Q3: STxM > STxF, p=0.0009
Contraction Frequency	OA: STxF > IF, p<0.0001<br>Q1: STxF > IF, p=0.0007<br>Q2: STxF > IF , p<0.0001<br>Q3: STxF > IF, p=0.005<br>Q4: STxF > IF, p=0.0047	N.S.	OA: IM > IF, p<0.0001<br>Q1,2,4: IM > IF, p<0.0001	N.S.

Supplemental Table 5b – 10 cm Bowel Outcomes Between Group Differences (STIM OFF)				
	Female	Male	Intact	Transected
Mean Amplitude (mmHg)	Q2: STxF > IF, p=0.0052	OA: IM > STxM, p=0.0099<br>Q1: IM > STxM, p=0.0317<br>Q2: IM > STxM, p=0.0256	OA: IM > IF, p<0.0001<br>Q1: IM > IF, p=0.0197<br>Q2: IM > IF, p<0.0001	N.S.
Maximum Amplitude (mmHg)	N.S.	OA: IM > STxM, p=0.005<br>Q1: IM > STxM, p=0.0276<br>Q2: IM > STxM, p=0.0073	OA: IM > IF, p=0.0006<br>Q2: IM > IF, p=0.0003	N.S.
Mean AUC (mmHg.sec)	OA: STxF > IF, p=0.0025<br>Q2: STxF > IF, p=0.0022<br>Q3: STxF > IF, p=0.0361	OA: IM > STxM, p=0.0368	OA: IM > IF, p<0.0001<br>Q1-4: IM > IF, p<0.0001	OA: STxM > STxF, p<0.0001<br>Q1,2: STxM > STxF, p<0.0001
Mean Duration (sec)	OA: IF > STxF, p=0.018<br>Q4: IF > STxF, p=0.0239	Q4: IM > STxM, p=0.0073	OA: IM > IF, p<0.0001<br>Q1-3: IM > IF, p<0.0001<br>Q4: IM > IF, p=0.0001	OA: STxM > STxF, p<0.0001<br>Q1-3: STxM > STxF, p<0.0001<br>Q4: STxM > STxF, p=0.0204
Mean Range	Q2: STxF> IF, p=0.0058	N.S.	OA: IM > IF, p<0.0001<br>Q1: IM > IF, p=0.0076<br>Q2: IM > IF, p<0.0001<br>Q4: IM > IF, p=0.04	N.S.
Contraction Count (within bouts)	OA: IF > STxF, p=0.0039<br>Q3: IF > STxF, p=0.0003	N.S.	Q1: IM > IF, p=0.0215<br>Q3: IF > IM, p=0.006	OA: STxM > STxF, p=0.0234<br>Q1: STxM > STxF, p=0.002
Contraction Count (non-bout)	OA: IF > STxF, p<0.0001<br>Q1: IF > STxF, p=0.0074<br>Q2: IF > STxF, p=0.0003<br>Q3,4: IF > STxF, p<0.0001	OA: IM > STxM, p<0.0001<br>Q1: IM > STxM, p=0.0318<br>Q2: IM > STxM, p<0.0001<br>Q3: IM > STxM, p=0.0186<br>Q4: IM > STxM, p=0.0001	Q2: IM > IF, p=0.0308	OA: STxM > STxF, p=0.0396<br>Q3: StxM > STxF, p=0.0277
Contraction Frequency	OA: STxF > IF, p<0.0001<br>Q1,2: STxF > IF, p<0.0001<br>Q3: STxF > IF, p=0.029<br>Q4: STxF > IF, p=0.0304	N.S.	OA: IM > IF, p<0.0001<br>Q1,2: IM > IF, p<0.0001<br>Q3: IM > IF, p=0.0017<br>Q4: IM > IF, p=0.0008	Q1: STxM > STxF, p=0.0287

Supplemental Table 6a – 10 cm Bowel Outcomes Within Group Differences (STIM ON)				
	Intact Female	Transected Female	Intact Male	Transected Male
Mean Amplitude (mmHg)	Q2 > Q1, p<0.0001<br>Q3 > Q1, p<0.0001<br>Q4 > Q1, p=0.0018<br>Q1: Off > On, p<0.0001	N.S.	N.S.	N.S.
Maximum Amplitude (mmHg)	Q2 > Q1, p<0.0001<br>Q3 > Q1, p<0.0001<br>Q4 > Q1, p=0.0216<br>Q3 > Q2, p=0.0214<br>Q3 > Q4, p=0.0173<br>OA: Off > On, p=0.0403<br>Q1: Off > On, p<0.0001	N.S.	N.S.	N.S.
Mean AUC (mmHg.sec)	Q2 > Q1, p=0.0017<br>Q3 > Q1, p=0.0009<br>Q4 > Q1, p=0.0163<br>Q1: Off > On, p=0.0003	N.S.	N.S.	N.S.
Mean Duration (sec)	N.S.	N.S.	N.S.	Q1 > Q3, p<0.0001<br>Q1 > Q4, p=0.004<br>Q2 > Q3, p<0.0001<br>Q2 > Q4, p=0.0065<br>Q3: Off > On, p<0.0001
Mean Range	Q2 > Q1, p<0.0001<br>Q3 > Q1, p<0.0001<br>Q4 > Q1, p=0.0019<br>Q1: Off > On, p<0.0001	N.S.	N.S.	N.S.
Contraction Count (within bouts)	OA: Off > On, p=0.0084<br>Q3: Off > On, p=0.0023	N.S.	Q1 > Q3, p=0.0403	N.S.
Contraction Count (non-bout)	Q3 > Q2, p=0.0485<br>OA: Off > On, p=0.0039<br>Q3: Off > On, p=0.0434<br>Q4: Off > On, p=0.002	N.S.	Q2 > Q1, p=0.0255	Q3 > Q1, p=0.0373<br>Q3 > Q4, p=0.0102
Contraction Frequency	Q3 > Q1, p=0.0014<br>Q3 > Q2, p=0.0014<br>Q3 > Q4, p=0.005	Q3 > Q1, p=0.0003<br>Q3 > Q2, p=0.0031<br>Q3 > Q4, p=0.0232<br>Q2: On > Off, p=0.0493<br>Q3: On > Off, p=0.0088	N.S.	Q3 > Q1, p=0.0183<br>Q3 > Q2, p=0.0163<br>Q3 > Q4, p=0.012<br>Q3: On > Off, p=0.0334

Supplemental Table 6b – 10 cm Bowel Outcomes Within Group Differences (STIM OFF)				
	Intact Female	Transected Female	Intact Male	Transected Male
Mean Amplitude (mmHg)	Q1 > Q2, p=0.0414<br>Q3 > Q2, p=0.0147	N.S.	N.S.	N.S.
Maximum Amplitude (mmHg)	Q3 > Q1, p=0.0312<br>Q3 > Q2, p=0.0038	N.S.	N.S.	N.S.
Mean AUC (mmHg.sec)	N.S.	N.S.	N.S.	N.S.
Mean Duration (sec)	N.S.	Q3 > Q1, p=0.0446<br>Q3 > Q4, p=0.0217	N.S.	Q1 > Q4, p=0.0008<br>Q2 > Q4, p=0.0002<br>Q3 > Q4, p=0.0002
Mean Range	Q3 > Q2, p=0.0405	N.S.	N.S.	N.S.
Contraction Count (within bouts)	Q3 > Q1, p=0.0002<br>Q3 > Q2, p=0.0006<br>Q3 > Q4, p=0.0095	Q2 > Q1, p=0.0498<br>Q2 > Q4, p=0.0293	Q4 > Q2, p=0.0466	N.S.
Contraction Count (non-bout)	Q3 > Q1, p=0.004<br>Q4 > Q1, p=0.0001<br>Q3 > Q2, p=0.0013<br>Q4 > Q2, p=0.0002	Q1 > Q4, p=0.0496	Q2 > Q1, p=0.0038<br>Q3 > Q1, p=0.0014<br>Q4 > Q1, p=0.0003	N.S.
Contraction Frequency	N.S.	N.S.	N.S.	N.S.
